# A framework for integrating biomedical knowledge in Wikidata with open biological and biomedical ontologies and MeSH keywords

**DOI:** 10.1016/j.heliyon.2024.e38448

**Published:** 2024-09-27

**Authors:** Houcemeddine Turki, Khalil Chebil, Bonaventure F.P. Dossou, Chris Chinenye Emezue, Abraham Toluwase Owodunni, Mohamed Ali Hadj Taieb, Mohamed Ben Aouicha

**Affiliations:** a Data Engineering and Semantics Research Unit, Faculty of Sciences of Sfax, University of Sfax, Sfax, Tunisia; b SisonkeBiotik Research Community, Johannesburg, South Africa; c Department of Computer Science, University of the People, Pasadena, CA, USA; d Department of Computer Science, College of Computer Engineering and Sciences, Prince Sattam Bin Abdulaziz University, Al Kharj 11942, Saudi Arabia; e Mila Quebec AI Institute, Montreal, Canada; f McGill University, Montreal, Canada; g Lelapa AI, Johannesburg, South Africa; h Technical University of Munich, Munich, Germany; i Faculty of Life Sciences, University of Ilorin, Ilorin, Nigeria

**Keywords:** Wikidata, Open biological and biomedical ontologies, MeSH keywords, Biomedical relation identification, Crowdsourcing, PubMed

## Abstract

This study presents a comprehensive framework to enhance Wikidata as an open and collaborative knowledge graph by integrating Open Biological and Biomedical Ontologies (OBO) and Medical Subject Headings (MeSH) keywords from PubMed publications. The primary data sources include OBO ontologies and MeSH keywords, which were collected and classified using SPARQL queries for RDF knowledge graphs. The semantic alignment between OBO ontologies and Wikidata was evaluated, revealing significant gaps and distorted representations that necessitate both automated and manual interventions for improvement. We employed pointwise mutual information to extract biomedical relations among the 5000 most common MeSH keywords in PubMed, achieving an accuracy of 89.40 % for superclass-based classification and 75.32 % for relation type-based classification. Additionally, Integrated Gradients were utilized to refine the classification by removing irrelevant MeSH qualifiers, enhancing overall efficiency. The framework also explored the use of MeSH keywords to identify PubMed reviews supporting unsupported Wikidata relations, finding that 45.8 % of these relations were not present in PubMed, indicating potential inconsistencies in Wikidata. The contributions of this study include improved methodologies for enriching Wikidata with biomedical information, validated semantic alignments, and efficient classification processes. This work enhances the interoperability and multilingual capabilities of biomedical ontologies and demonstrates the critical role of MeSH keywords in verifying semantic relations, thereby contributing to the robustness and accuracy of collaborative biomedical knowledge graphs.

## 1 Introduction

Currently, open and collaborative knowledge graphs are becoming a pillar for the practice of information fusion [1]. Due to their structured format in the Resource Description Framework (10.13039/100012280RDF) Format and their flexible data model, they can be easily extended to support multiple topics and integrate external resources [1]. In this context, multiple works have been done to recursively enrich knowledge graphs using knowledge graph-driven information retrieval from scientific texts [2]. Several efforts have also been made to automate the exchange of semantic information between knowledge graphs and ontologies [3]. Created in October 2012, Wikidata is a large-scale open and collaborative multilingual knowledge graph that was initially developed to support Wikipedia with structured data but has grown in use to cover various disciplines ranging from cultural heritage [4] and linguistics [5] to medicine [1].

Thanks to several properties, Wikidata has already been aligned to multiple external resources [6]. These external resources include authority control databases like VIAF [7], movie catalogs like IMDb [8], molecular biology databases like RefSeq [9], and most importantly the Open Biological and Biomedical Ontologies (OBO), a set of ontologies related to medical knowledge and released online under open licenses like CC-BY and CC0 [10]. The latter has been linked to Wikidata particularly thanks to the Gene Wiki Initiative [9]. Currently, many OBO ontologies are not only linked to Wikidata using external identifier statements but also used to enrich Wikidata with biomedical knowledge such as the *Human Disease Ontology* [11], the *Gene Ontology* [12], and the *Evidence and Conclusion Ontology* [10]. Beyond this, due to the lack of coverage of all the aspects of biomedical information in open biological and biomedical ontologies, there have been significant efforts to develop methods to retrieve semantic information from biomedical scholarly publications to enrich Wikidata [13]. Most of these methods are based on machine learning techniques [13]. Another direction in the same context was the usage of bibliographic metadata, particularly controlled keywords such as the Medical Subject Headings (MeSH) keywords of the PubMed scholarly publications, to classify biomedical relations in Wikidata (so-called *MeSH2Matrix*) [14].

In this research work, we propose a framework for enriching biomedical information in Wikidata as an open and collaborative knowledge graph by using the MeSH Keywords of PubMed scholarly publications and the semantic alignment of Wikidata items to entities in open biological and biomedical ontologies. We also propose an approach to enhance the semantic information of open knowledge graphs based on the statements about corresponding items in Wikidata.

The paper is structured into six sections. The first section provides an overview of open semantic resources in healthcare, focusing on their use in enhancing biomedical knowledge graphs like Wikidata, and examines the strengths and limitations of resources such as UMLS, BioPortal, and SNOMED CT (Section 2.1). The second section discusses Wikidata as a large-scale biomedical knowledge graph, exploring challenges and opportunities in aligning it with Open Biological and Biomedical Ontologies (OBO) and addressing technical and licensing issues for seamless integration (Section 2.2). The third section covers the state-of-the-art in biomedical relation extraction and classification, focusing on methods for extracting and classifying relations from MeSH keywords in PubMed and the role of machine learning techniques (Section 2.3). The fourth section addresses knowledge graph-based ontology engineering, discussing best practices for integrating Wikidata and OBO ontologies and leveraging crowdsourcing and human validation (Section 2.4). The fifth section explains our approach for integrating biomedical knowledge into Wikidata using MeSH Keywords and OBO ontologies, investigating the impact on data robustness, accuracy, interoperability, and multilingual capabilities (Section 3). The sixth section presents the results of the proposed methods and compares them with previous research (Section 4). Finally, the paper concludes with future directions, summarizing findings and suggesting avenues for further research (Section 5).

## 2 Related work

### 2.1 Open semantic resources in healthcare

In the realm of healthcare, open semantic resources have emerged as powerful tools, utilizing technologies such as ontologies and linked data to organize and represent medical knowledge in machine-readable formats [15]. These resources hold immense potential for enhancing patient care, driving medical research, and fostering innovation within the healthcare sector [15]. One significant initiative in this domain is the Unified Medical Language System (UMLS), developed by the National Library of Medicine (NLM) [16]. UMLS integrates diverse biomedical terminologies, including the International Classification of Diseases (ICD), into a unified semantic framework, facilitating interoperability between healthcare systems and supporting applications such as natural language processing and clinical decision support [16]. Another pivotal resource is BioPortal, serving as a repository for open biomedical ontologies [17]. This platform promotes collaboration and innovation in biomedical informatics by providing a centralized hub for sharing and accessing ontological resources, thereby advancing healthcare analytics and research [17].

SNOMED CT stands out as a comprehensive clinical terminology system, facilitating standardized communication across healthcare domains [18]. Its open development model ensures relevance to evolving medical practices, enabling precise data exchange and analysis within the healthcare community [18]. The SIB Swiss Institute of Bioinformatics Semantic Web of Data is another noteworthy platform offering access to a diverse array of biomedical datasets and ontologies [19]. By enabling interdisciplinary research and knowledge discovery in biomedicine through semantic structuring, this platform contributes significantly to the advancement of healthcare [19]. UniProt Knowledgebase provides open access to protein sequence and functional information, supporting researchers in exploring the functional properties of proteins and their implications in biological processes through semantic technologies [20]. YummyData serves as an open platform for sharing and discovering linked data resources across healthcare and life sciences domains, fostering collaboration and knowledge exchange among stakeholders [21]. Additional initiatives such as the International Classification of Functioning, Disabilities and Health (ICF) [22], the Ontology for Biomedical Investigations (OBI) [23], and Semantic DICOM [24] further illustrate the breadth and depth of open semantic resources available in medical practice. These initiatives provide standardized vocabularies and frameworks for assessing functioning, describing biomedical investigations, and enhancing interoperability in medical imaging, respectively.

Moreover, the creation of such resources often involves methodologies like Bio2RDF, which converts biological databases into RDF format, enabling integration and exploration of data from multiple sources for biomedical research, drug discovery, and personalized medicine [25]. Additionally, the development of data models, such as those utilized by OHDSI for standardizing and harmonizing electronic health record data, plays a crucial role in ensuring interoperability and facilitating large-scale observational studies and comparative effectiveness research across diverse healthcare settings [26]. Furthermore, the use of machine learning techniques has become increasingly prevalent in developing biomedical semantic resources [27]. Machine learning algorithms can analyze vast amounts of biomedical data to identify patterns and relationships, aiding in the creation and refinement of ontologies and terminologies [27]. These techniques contribute to the automation of ontology development, improving the accuracy and efficiency of semantic resource creation in the biomedical domain [27]. Additionally, the Open Biological and Biomedical Ontologies (OBO) Foundry serves as a community-driven effort to develop and maintain a suite of interoperable ontologies in the biomedical domain [28]. The OBO Foundry principles promote collaboration, openness, and adherence to best practices in ontology development, ensuring that ontologies are well-structured, interoperable, and widely adopted within the biomedical research community [28].

### 2.2 Wikidata as a large-scale knowledge graph for integrating biomedical information

**Fig. 1:**
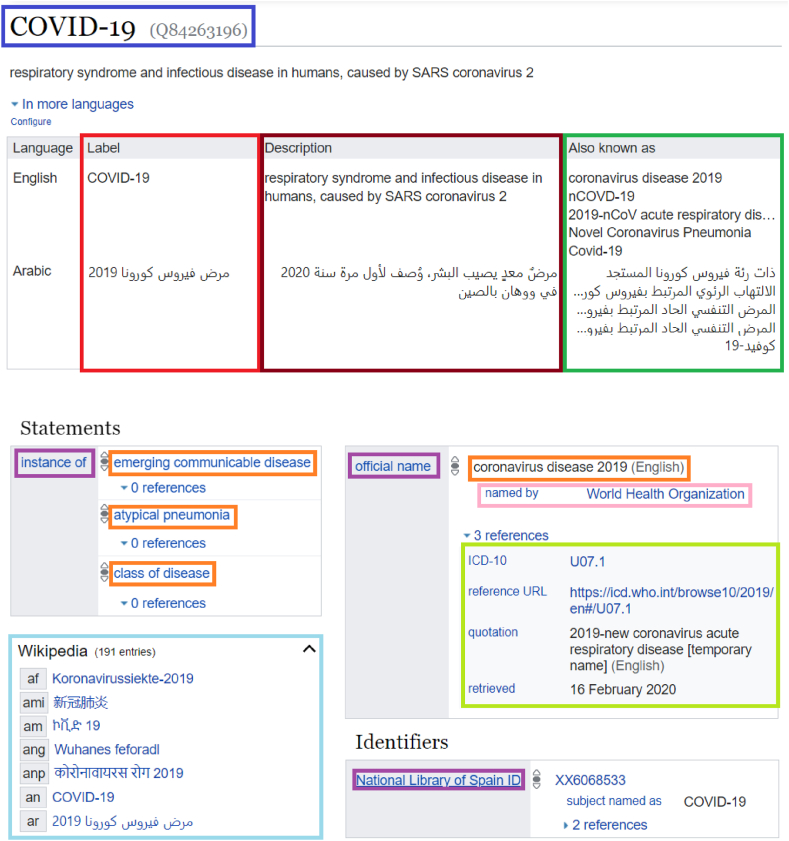
The representation of a Wikidata item.

As a knowledge graph, Wikidata (https://www.wikidata.org) includes semantic information about various kinds of concepts. Such concepts include diseases, drugs, anatomical structures, chemical compounds, genes, and proteins among other kinds of entities [1,8]. When describing a concept, it is common to use statements in the form of triples. In such a triple, the subject represents the entity being described (Blue in Fig. 1), the predicate indicates the type of statement being made (Purple in Fig. 1), and the object (Orange in Fig. 1) can take various forms, such as another entity, an external identifier, a URL, a value, a datetime, and more [1,8]. Every concept in the Wikidata knowledge base is identified by a Q-number that serves as a language-independent identifier. For instance, the concept of a disease is identified by the Q-number [Q12136] (Blue in Fig. 1). On the other hand, every type of statement in Wikidata is represented by a property that has a P-number as a language-independent identifier, such as the property 'instance of' [P31]. Both items and properties are given labels (*rdfs:label*, Red in Fig. 1), descriptions (*schema:description*, Brown in Fig. 1), and aliases (*skos:altLabel*, Blue in Fig. 1) in multiple natural languages, as documented in various studies [1,8]. Moreover, every statement in Wikidata can be verified through a reference system that takes the form of triples (Light Green in Fig. 1), which ensures its accuracy, much like fact-checking in Wikipedia [1,8].

[Source: https://www.wikidata.org/wiki/Q84263196]: Subject (Blue), Labels (Red), Descriptions (Brown), Aliases (Green), Predicates (Purple), Objects (Orange), Qualifiers (Pink), References (Light Green), and Semantic Alignments to Wikipedia (Light Blue).

Given its structured format, Wikidata can be parsed using a variety of tools [29]. It has its SPARQL endpoint (https://query.wikidata.org) that allows users to extract semantic information from Wikidata based on a set of conditions implemented in SPARQL, a query language for knowledge graphs [29]. It also has an application programming interface (*MediaWiki API*) that allows editing and getting information from Wikidata^2^ programmatically [29]. This API can be handled using a user-friendly Python Library called *Wikibase Integrator*.^3^ Wikidata also has Resource Description Framework (RDF) dumps^4^ that can be downloaded to allow the offline processing of the database. There is also a web tool called Mix'n’match^5^ for the semi-automatic semantic alignment between Wikidata items and entities in external resources [30]. These four resources have already been used for crowdsourcing, reusing, and automatically enriching medical information in Wikidata.

SPARQL has been used to create real-time dashboards and web tools for clinical decision support [1]. SPARQL has also been used for implementing logical constraints to validate relational and non-relational statements about the medical domain [31]. MediaWiki API has been used to mass import biomedical knowledge from open resources, particularly Open Biological and Biomedical Ontologies (OBO) like *Gene Ontology* and National Center for Biotechnology Information (NCBI) resources like *RefSeq* to Wikidata [9]. Wikidata dumps have been processed using graph learning techniques for identifying implicit relations in the knowledge graph and explicitly declaring them in Wikidata [32]. Mix'n’match has been used to assign external identifiers from OBO ontologies and NCBI resources to corresponding biomedical items in Wikidata allowing the federated use of Wikidata with other biomedical databases for driving medical applications as well as the cross-enrichment of Wikidata and other linked biomedical resources [33].

In the interaction between Wikidata and the Open Biological and Biomedical Ontology (OBO) framework, various properties and items play significant roles, influencing how ontologies are shared, integrated, and evolved across both platforms. Firstly, ontologies from the OBO framework are represented in Wikidata as instances of the “*Ontology*” class (Q324254) using the “*instance of*” (P31) property. This distinguishes them within the Wikidata ecosystem. They are further linked to larger collections or initiatives such as the *Open Biological and Biomedical Ontology Foundry* (OBO Foundry) (Q4117183) using the “*part of*” (P361) property. Ontologies undergo updates or releases, which are tracked using the “*has edition or translation*” (P747) property in Wikidata, allowing users to monitor their evolution over time. Contributors to the development or maintenance of the ontology are acknowledged through the “*contributor to the creative work or subject*” (P767) property. To ensure interoperability, ontologies are linked to their unique identifiers (URIs) using the “*exact match*” (P2888) property.

Finally, ontologies are linked to their corresponding Wikidata property for concept IDs using the “*Wikidata Property*” (P1687) property, facilitating knowledge sharing and integration within the biomedical and biological research communities. When considering license stacking, the original license of the imported ontology is retained and documented in Wikidata using the “*copyright license*” (P275) property. This ensures that the terms of use established by the ontology's creators are respected and acknowledged. Regarding versioning, Wikidata tracks ontology versions using the “*has edition or translation*” (P747) property, enabling users to access and reference specific releases of the ontology. This aligns with OBO's versioning practices, ensuring that users have access to the most up-to-date and accurate ontology data for reproducibility and continuity in research.

### 2.3 Biomedical relation extraction and classification

Biomedical relations have always been extracted and classified based on plain or annotated texts [34,35]. Most of these algorithms developed for such a purpose cover a limited number of relation types [34]. These relation types are mainly drug-disease relations, drug-drug interactions, and drug adverse effects [35,36]. Analyzed texts include online encyclopedias like *Wikipedia* [37], open-access repositories like *PubMed Central* [35], and the titles and abstracts of scholarly publications as available in bibliographic databases such as *PubMed* [38]. Classical methods for the identification and classification of biomedical relations involved the named entity annotation of the analyzed texts using semantic resources like *Medical Subject Headings* (MeSH) or *Unified Medical Language System* (UMLS) Metathesaurus [38]. Then, the semantic relations are identified and classified using term co-occurrence analysis based on clustering methods like Latent Dirichlet Allocation (LDA) [38], rule-based approaches such as parse tree analysis and lexical pattern analysis [34] and probabilistic metrics like term frequency and inverse document frequency (TF-IDF) and semantic similarity measures (SemSim) [38].

With the rise of machine learning, new techniques have emerged for a more efficient extraction and classification of biomedical relations [35,36]. The recognition and classification of the association between terms becomes based on Word and Positional Embeddings like Word2Vec and GloVe as well as on pre-trained language models like BERT [36,39]. The combination of a Long Short Term Memory (LSTM) network and other machine-learning algorithms such as attention network, convolutional neural network (CNN) or Conditional random field (CRF) has also been proven as efficient in extracting and classifying biomedical relations [35,36]. Most of the training of such machine-learning algorithms is done thanks to distant supervision where an existing semantic database like open knowledge graphs is used to collect samples for the relations to be extracted [40].

The biomedical relation extraction and classification task could be easier if the analyzed dataset is the set of controlled keywords for the considered scholarly publications [14]. As an example, Medical Subject Headings (MeSH) Keywords in PubMed are structured keywords with a Heading/Qualifier pattern where the heading (e.g., *Hepatitis C*) is the main topic of the paper and the qualifier (e.g., *Drug Therapy*) is the facet of the heading evoked by the paper [14]. Due to their structured format and their derivation from the MeSH taxonomy, MeSH keywords can be analyzed with minor pre-processing to provide significant insights into biomedical knowledge. Co-occurrence analysis of the MeSH keywords has successfully identified the mechanisms behind drug-drug interactions [41]. As well, the analysis of the qualifiers of two co-occurring MeSH terms has been successful in classifying biomedical associations using Wikidata as a benchmark of classified semantic relations [14].

### 2.4 Knowledge graph-based ontology engineering

During the last few years, ontology engineering has evolved to transform the practices of the semantic web community [42]. This includes the creation of user-friendly ontology editors like Protégé, the development of methods for intrinsic data modeling, processing, and validation, and the update of the semantic web standards like *Web Ontology Language* (OWL) and *RDF Schema* (RDFS) to ensure the interoperability of ontologies with other semantic resources, particularly open knowledge graphs [42]. With the development of open knowledge graphs like Wikidata, the interaction between ontologies and knowledge graphs has become one of the most efficient methods for augmenting semantic data in ontologies [3]. The principle is to make an alignment between ontology items and knowledge graph items, and then mass import the semantic knowledge inside the considered ontology into the knowledge graph [3].

Then, as knowledge graphs, particularly Wikidata, evolve through human edits and crowdsourcing from multiple sources, new statements about the items covered by the ontology are added to knowledge graphs [1]. These new semantic claims can be retrieved from the knowledge graph and used to enrich the source ontology [3,9]. Another less common application of knowledge graphs for ontology engineering is to derive the backbone of an ontology from the data model of a knowledge graph [43,44]. This can happen at the beginning of the conception of the ontology through parsing a specific part of the source knowledge graph [43]. This can also occur in post-production by using centrality analysis on knowledge graphs to restrict the ontology to its most important items and semantic relations (so-called ontology summarization) [44].

### 2.5 Biomedical relation extraction from PubMed

Biomedical Relation Extraction (BRE) plays a pivotal role in discerning and categorizing connections between entities mentioned in biomedical text, crucial for various applications in life sciences like constructing knowledge graphs, facilitating drug discovery, and unraveling disease mechanisms. PubMed, an invaluable repository of biomedical literature, stands as a cornerstone resource for BRE tasks owing to its extensive array of scientific publications. Typically, BRE focuses on processing abstracts of scholarly works using various methodologies.

Some methodologies, particularly those rooted in supervised learning, have gained prominence. For instance, Liu et al. [45] propose an innovative deep learning architecture employing attention mechanisms to grasp long-range dependencies within sentences, thereby enhancing relation extraction accuracy. Meanwhile, Peng et al. [46] introduce a cross-sentence N-ary relation extraction method utilizing dependency parsing trees and support vector machine classifiers to identify intricate relations spanning multiple sentences. In a similar vein, Xu et al. [47] present a support vector machine-based approach incorporating domain-specific features and sentence structure information for relation extraction. Moreover, Peng et al. [48] develop a comprehensive deep learning framework combining support vector machines, convolutional neural networks, and recurrent neural networks for chemical-protein relation extraction.

Despite the prevalence of supervised learning in biomedical relation extraction from PubMed, alternative approaches such as unsupervised or semi-supervised learning and language models have garnered attention. Song et al. [49] explore rule-based and dictionary-based methods for relation extraction from PubMed abstracts, while Alam et al. [50] propose a pattern-based approach for extracting chemical-disease relations from biomedical literature. Furthermore, Alimova et al. [36] delve into the efficacy of sentence embedding techniques in enhancing relation extraction model performance. Sousa et al. [51] underscore the significance of domain-specific knowledge and resources in refining the accuracy of biomedical relation extraction systems.

These studies underscore significant strides in biomedical relation extraction from PubMed data. However, there exists ample scope for further enhancement by exploring novel methodologies harnessing bibliographic metadata beyond abstracts. For instance, MeSH keywords hold promise in directly informing biomedical relation extraction methods beyond their conventional applications in retrieving unclassified relations [52] and augmenting MeSH indexing [53].

### 2.6 Challenges

While substantial progress has been made in leveraging open semantic resources and knowledge graphs to enhance biomedical information, several challenges persist that justify the need for continued research in this area. One significant challenge is the accurate and efficient alignment of entities across different semantic resources. The heterogeneity in data models, terminologies, and formats poses a barrier to seamless integration and interoperability. Moreover, the dynamic nature of biomedical knowledge necessitates continuous updates and validation of the integrated data to ensure its relevance and accuracy.

Another challenge lies in the extraction and classification of biomedical relations from vast and diverse datasets. Although machine learning and deep learning techniques have improved the efficiency of relation extraction, the dependency on large annotated datasets for training remains a constraint. The complexity of biomedical texts, characterized by domain-specific jargon and implicit relationships, further complicates the extraction process. Additionally, the need for robust mechanisms to handle multilingual data and ensure the quality and reliability of extracted information remains a critical area of concern.

The literature reviewed highlights these challenges and underscores the importance of developing innovative solutions to address them. By proposing a framework that utilizes MeSH keywords and semantic alignment with open biomedical ontologies, this research aims to enhance the semantic richness and interoperability of biomedical knowledge graphs like Wikidata. This approach not only seeks to improve data robustness and accuracy but also addresses the multilingual capabilities essential for global biomedical research.

The outcome of this literature review confirms the necessity of our proposed framework and validates its potential to overcome the identified challenges. By addressing the integration, extraction, and classification issues, this work aims to contribute significantly to the field of biomedical informatics, ultimately facilitating better data-driven decision-making and fostering advancements in medical research and healthcare.

## 3 Proposed approach

Our research design involves a combination of qualitative and quantitative methods, including literature review, data collection and preprocessing, semantic alignment analysis, machine learning techniques, validation, and impact assessment. Our approach is primarily based on the interaction between Wikidata and Open Biological and Biomedical Ontologies on one hand, and Wikidata and MeSH Keywords on the other hand. The exchange between Wikidata and OBO involves validating the semantic alignment between Wikidata items and entities in open biomedical ontologies to identify missing ontology items and corrupted external identifiers in Wikidata. It also includes identifying Wikidata relations and multilingual names and descriptions for the items included in the ontology. The interaction between Wikidata and MeSH Keywords is based on analyzing the association of MeSH Keywords in PubMed scholarly publications. This involves identifying and classifying semantic relations from MeSH keyword co-occurrences and identifying references from PubMed that confirm unsupported statements in Wikidata. The output of our approach will be stored in datasets for human validation before finally being added to OBO or Wikidata. This comprehensive approach aims to significantly enhance Wikidata's biomedical knowledge, improving its accuracy, interoperability, and practical utility for biomedical research and clinical decision-making.

Human validation combined with automation presents a potent strategy for enhancing the quality of knowledge graphs, which serve as the foundation for various applications in semantic search, recommendation systems, and AI [54]. By integrating human validation into automated processes, several key benefits emerge. Firstly, it improves accuracy by addressing contextual nuances and language ambiguities that automated methods may overlook [55]. Secondly, human intervention is essential for error correction, detecting and rectifying mistakes introduced by automated processes [56]. Thirdly, humans can provide semantic validation, ensuring that the relationships within the knowledge graph align with the broader domain context [57]. Additionally, human validators contribute to data completeness by identifying missing elements that automated tools may overlook [58]. Furthermore, humans offer flexibility in adapting to evolving domains and updating the knowledge graph accordingly [57]. Finally, human validation serves as a critical quality assurance step, ensuring that the knowledge graph meets the required standards, particularly in applications where errors could have significant consequences [55].

### 3.1 Interaction between Wikidata and Open Biological and Biomedical Ontologies

This approach begins by extracting the list of OBO ontologies with a valid URI and a Wikidata property that brings the ID of a Wikidata item in a given OBO ontology as an external identifier using a SPARQL query (Query T1). In Wikidata, open biological and biomedical ontologies are defined as instances of [P31] ontology [Q324254] and part of [P361] Open Biological and Biomedical Ontologies [Q4117183]. They are linked to their Uniform Resource Identifier (URI) using the *exact match* [P2888] property and to their corresponding external identifier property using *Wikidata property* [P1687] statements. We manually eliminated the ontology with invalid URI or OWL formatting issues.

Next, we retrieved the items having an ID in a given ontology from Wikidata using another SPARQL query (Query T2). This SPARQL query retrieves all items (?subject) from a specified ontology that possesses an external identifier (e.g., P699 for *Disease Ontology ID*), along with the assigned value stored in ?subjectID. The URI of the same ontology was used to identify the IDs of all the entities in it using Pronto, a Python Library to parse open biological and biomedical ontologies.^6^ The final two lists were compared to identify missing items and obsolete identifiers in Wikidata. After verifying the semantic alignment between the two knowledge resources, we formulated a SPARQL query to extract the labels (*rdfs:label*), descriptions (*schema:description*), and aliases (*skos:altLabel*) of every Wikidata item corresponding to an ontology entity (Query T3). The query initially identifies all items (?subject) with an external identifier within a designated ontology (?subjectID). Subsequently, it utilizes the VALUES clause to define the properties for retrieval: rdfs:label (preferred label), schema:description (description), and skos:altLabel (alternative label). Lastly, it retrieves the object values for each property alongside their respective languages, utilizing the LANG function to specify the language of each object. Additionally, we used another SPARQL query to extract the semantic relations between the ontology entities and other entities from the same ontology or other OBO ontologies, as available in Wikidata (Query T4). The SPARQL query first identifies all items (?subject) with an identifier (?subjectID) in a specific ontology. Then, it locates all objects (?object) with an identifier (?objectID) in a different ontology. Finally, it retrieves the Wikidata property (?prop) that links every item from the first ontology to every item from the second ontology, if such a property exists. Such semantic knowledge can be proposed to the curators of the target ontology for ontology enrichment purposes. The SPARQL queries for this part are featured in Supplementary Table S1. For T3 and T4, we only consider the ontologies having 100 valid identifiers or more in Wikidata.

### 3.2 Biomedical relation extraction from MeSH keywords

In order to extract biomedical relations from MeSH Keywords, we start by extracting the list of all the Wikidata items corresponding to MeSH keywords thanks to a SPARQL query (Query L1). This is enabled thanks to the *MeSH descriptor ID* [P486] Wikidata statements. The query selects ?subject and ?subjectID where ?subject has a MeSH identifier (wdt:P486) represented by ?subjectID. After that, we use BioPython, a Python Library to parse NCBI Entrez API [59], to identify the 5000 top MeSH keywords in PubMed. Then, we use BioPython again to compute the semantic relationship between every couple of the 5000 considered MeSH keywords based on pointwise mutual information (PMI). Let *x* and *y* be two MeSH keywords, *R* the number of scholarly publications in PubMed, *N(x,y)* the number of search results for the association of *x* and *y* in PubMed scholarly publications, and *N(x)* and *N(y)* be the respective the number of search results for *x* and *y* as MeSH keywords in PubMed scholarly publications, the equation of the PMI is defined in Equation (1) [[60], [61]]:

$$If\;N\left(x,y\right)\;>0:PMI\left(x,y\right)=log_{2}\left(\frac{N\left(x,y\right).R}{N\left(x\right).N\left(y\right)}\right)=log_{2}\left(N\left(x,y\right)\right)+log_{2}\left(R\right)-log_{2}\left(N\left(x\right)\right)-log_{2}\left(N\left(y\right)\right)$$

(1) IfN(x,y)=0:PMI(x,y)=(−10)

To define the threshold for PMI, we extract the list of Wikidata relations between all MeSH keywords using another SPARQL query (Query L2). The query selects ?subject, ?subjectID, ?prop, ?object, and ?objectID. It finds triples where both ?subject and ?object have MeSH identifiers (wdt:P486) represented by ?subjectID and ?objectID respectively. It then retrieves all triples where there is a relation (?prop) between the MeSH terms represented by ?subject and ?object. We set the threshold by rounding down the Pointwise Mutual Information (PMI) values to the nearest whole number for the semantic relations between MeSH terms sourced from Wikidata. The threshold is the mode of the rounded PMI values (i.e., the rounded value of PMI with the highest frequency among Wikidata semantic relations). The Wikidata relations below the PMI threshold are flagged for human validation. The associations between MeSH keywords achieving a PMI superior or equal to the threshold are kept as valid relations to be added to Wikidata after classification.

### 3.3 Biomedical relation classification based on MeSH keywords

**Fig. 2:**
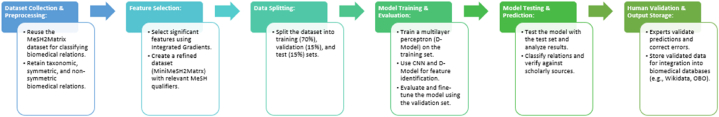
Pipeline for the classification of biomedical relations using MeSH qualifiers.

This stage (Fig. 2) is based on *MeSH2Matrix,*^7^ a dataset for the classification of biomedical relations based on MeSH qualifiers [14]. The dataset includes a set of matrices of correspondence between the qualifiers of the subjects and objects of Wikidata semantic relations annotated by the Wikidata IDs of corresponding relation types. Each matrix evaluates the frequency of MeSH qualifier assignment (76 qualifiers) to the subject and object of the analyzed relation. We filter the dataset to discard non-biomedical relations and keep *taxonomic relations*, *biomedical symmetric relations*, and *biomedical non-symmetric relations*. This is mainly due to the lack of ability of the MeSH qualifier-based classification to discriminate between non-biomedical relations [14]. Then, we reduce the dimensions of the matrices by only considering the MeSH qualifiers that contribute to biomedical relation classification based on the Integrated Gradients method [14].

Integrated Gradients is a technique in explainable AI that assesses how each input feature affects a model's output [62]. It computes the integral of gradients along a path from a baseline to the input, attributing scores to each feature based on its influence on the model's prediction [62]. This method aids in understanding model decisions, debugging, bias detection, and enhancing transparency across different domains [62]. To apply the Integrated Gradients approach in MeSH2Matrix, we first trained the dataset employing both a convolutional neural network and a dense model, as outlined in Turki et al. [14]. Following the training, we identified the MeSH qualifiers that ranked among the top ten most significant features for attributing matrices to each superclass, based on Integrated Gradients' computations from one of the aforementioned models [14]. We consequently keep thirty MeSH qualifiers^8^: *analysis*, *pathology*, *diagnosis*, *etiology*, *complications*, *metabolism*, *biosynthesis*, *therapy*, *drug therapy*, *methods*, *chemistry*, *diagnostic imaging*, *genetics*, *physiopathology*, *epidemiology*, *blood*, *pharmacology*, *analogs & derivatives*, *adverse effects*, *therapeutic use*, *surgery*, *pharmacokinetics*, *agonists*, *ethnology*, *administration & dosage*, *drug effects*, *enzymology*, *physiology*, *toxicity*, and *immunology*. The resulting dataset, called *MiniMeSH2Matrix*, will cover 11,961 *taxonomic* relations (3 relation types), 801 *biomedical symmetric* relations (3 relation types), and 17,931 *biomedical non-symmetric* relations (50 relation types), with a total coverage of 30,693 distinct 30∗30 matrices (56 relation types, 3 superclasses) [14].

The MiniMeSH2Matrix dataset will be divided into three sets for machine-learning model training and evaluation. The training set will represent 70 % of the matrices (21,485 matrices). The dev set, also known as the evaluation set, will include 15 % of the matrices (4063 matrices). The remaining 15 % of the matrices (4063 matrices), will be included in the test set. To classify biomedical relations, we will use a multilayer perceptron called D-Model. This model consists of an input layer with an output feature size of 900, a hidden layer of 450, and an output layer with an output feature size equal to the number of classes (59 for specific relation classification and 3 for metaclass-based relation classification [14]. The model will be trained to classify the relations into three superclasses: *Taxonomic*, *Symmetric*, and *Non-Symmetric*. Then, it will be trained to classify the relations into 59 relation types. The algorithm will be evaluated using two statistical metrics: *Accuracy* (Equation (2)) and *F1-Score* (Equation (3)) [63].

(2) $$Accuracy=\frac{TP+TN}{TP+FP+TN+FN}$$

(3) $$F1=\frac{2\;*\;Precision\;*\;Recall}{Precision+Recall}$$

The Accuracy is based on four basic measures [63]: *True Positive* refers to the number of elements accurately matched to their respective classes, while *True Negative* is the number of elements accurately not attributed to unrelated classes, *False Positive* is the number of elements misassigned to unrelated classes, and False Negative is the number of elements mistakenly not linked to their respective classes. The F1-Score is a combination of Precision ($\frac{TP}{TP+FP}$) and Recall ($\frac{TP}{TP+FN}$) [63]. A confusion matrix will also be traced based on test data for the superclass-based classification to study the dynamics of the algorithm.

Later, the algorithm will be used to classify the relevant associations between MeSH Keywords (having *PMI* superior or equal to the threshold) through the analysis of a maximum of twenty scholarly publications for every semantic relation. The analyzed publications should incorporate MeSH Keywords as major topics and include MeSH qualifiers for the considered subjects and objects.^9^ A relation type is only assigned to an association if it is identified by the relation type-based classification and its category is accurately attributed to the relation by the superclass-based classification. The output of the supervised classification will be stored in a dataset. To evaluate the efficiency of the agreement between the relation type-based classification and the superclass-based classification, we trace another confusion matrix that compares the correct assignment of labels in relation type-based classification to the one in superclass-based classification based on test data.

### 3.4 Adding references to unsupported relations in wikidata based on MeSH keywords

This part begins by identifying random 1000 Wikidata relations between MeSH items lacking references using a SPARQL query (Query L3). The query finds triples where both ?subject and ?object have MeSH identifiers (wdt:P486) represented by ?subjectID and ?objectID respectively. It then filters out properties (?prop) that are not direct Wikidata properties and retrieves statements (?statement) related to the MeSH items. It further filters out statements that have references by checking if there is no prov:wasDerivedFrom property associated with the statement. Finally, it limits the results to 1000 entries. Next, using the BioPython Python Library, we will explore the associations between the subjects and objects of the semantic relations in the MeSH keywords of recent (last five years) PubMed scholarly publications with a high level of evidence (i.e., Review). In the realm of Evidence-Based Medicine, prominent sources for clinical guidance include systematic reviews, high-level guidelines, and meta-analyses. These sources distill and synthesize scholarly literature, forming a cornerstone of clinical practice reference [64]. To pinpoint pertinent references for a given semantic relation, we identify the top five PubMed search results according to the *Best Match* sorting algorithm [65].

The Best Match sorting algorithm is a machine-learning-based ranking model using *LambdaMART*, a technique that transforms the ranking of search engine results into a pairwise classification or regression task [66], and *BM25*, a bag-of-words retrieval function that quantifies how many documents are relevant to a search query [67], to find the publications that mostly match to a PubMed search query [65]. The PubMed IDs of the references are stored in a preliminary list of references for unsupported Wikidata statements. Our study utilized the PubMed search engine to identify references related to the Wikidata semantic relations between MeSH terms. We analyzed unsupported relations that were not linked to a PubMed publication, as well as those linked to three or more PubMed publications. The following results and discussion are based on this analysis.

## 4 Results and discussion

Our work involves a two-way interaction between Wikidata and Open Biological and Biomedical Ontologies, as explained in Section 4.1. Additionally, we enrich Wikidata with biomedical relations using MeSH Keywords and provide relevant references to them from PubMed, as described in Section 4.2.

### 4.1 Interaction between Wikidata and Open Biological and Biomedical Ontologies

As of August 8th, 2023, we identified twenty OBO ontologies that are linked to Wikidata and have a URI statement. From these ontologies, fifteen are valid as shown in Table 1. This number is limited when compared to the overall number of OBO ontologies represented as Wikidata items (182 ontologies), meaning that further efforts should be provided to better represent Open Biological and Biomedical Ontologies in Wikidata [68]. The ontologies having both Wikidata properties and URI statements (Table 1) cover molecular biology data like *Gene Ontology* and *Sequence types and features ontology*, upper-level data like *Relations Ontology*, taxon and anatomical structure data like *Uberon* and *Cell Line Ontology*, and clinical medicine data like *Disease Ontology* and *Vaccine Ontology*. This is a limited part of the ontologies available at OBO Foundry [28]. On the same day, there are 201 distinct OBO ontologies^10^, also covering agriculture, chemistry, and pharmacology among other domains [28]. Some of the missing ontologies have a Wikidata item such as *Chemical Methods Ontology* [Q55118301] However, they do not have Wikidata properties. Further efforts should be made to increase the alignment between Wikidata and OBO ontologies. This can be done by proposing new properties for the missing OBO ontologies^11^ [1] and the use of tools like Mix'n’match to add OBO ontology IDs to Wikidata items [30].

**Table 1:** Valid OBO Ontologies linked to Wikidata (as of August 8th, 2023).

Ontology	Description	URI	Wikidata Property	License
*Sequence types and features ontology* [Q81661803]	An ontology for annotation of sequences	http://purl.obolibrary.org/obo/so.owl	*Sequence Ontology ID* [P3986]	CC-BY 4.0
*Symptom Ontology* [Q81661810]	An ontology of disease symptoms	http://purl.obolibrary.org/obo/symp.owl	*Symptom Ontology ID* [P8656]	CC0
*Gene Ontology* [Q135085]	An ontology for describing the function of genes and gene products	http://purl.obolibrary.org/obo/go.owl	*Gene Ontology ID* [P686]	CC-BY 4.0
*Disease Ontology* [Q5282129]	A formal ontology of human disease	http://purl.obolibrary.org/obo/doid.owl	*Disease Ontology ID* [P699]	CC0
*Uberon* [Q7876491]	A comparative anatomy ontology representing a variety of structures found in animals, such as lungs, muscles, bones, feathers, and fins	http://purl.obolibrary.org/obo/uberon.owl	UBERON ID [P1554]	CC-BY 3.0
*Human Phenotype Ontology* [Q17027854]	A logical standard to describe and computationally analyze phenotypic abnormalities found in human disease	http://purl.obolibrary.org/obo/hp.owl	*Human Phenotype Ontology ID* [P3841]	HPO
*Cell line Ontology* [Q21039006]	An ontology that describes the anatomic origin and nature of eukaryotic cell lines	http://purl.obolibrary.org/obo/clo.owl	*Cell Line Ontology ID* [P2158]	CC-BY 3.0
*Mondo Disease Ontology* [Q27468140]	An ontology for the integration of information on cross-species diseases	http://purl.obolibrary.org/obo/mondo.owl	*Mondo ID* [P5270]	CC-BY 4.0
*Evidence and Conclusion Ontology* [Q28445410]	An ontology that supports assertions about things (such as scientific conclusions, gene annotations, or other statements of fact) that result from scientific research	http://purl.obolibrary.org/obo/eco.owl	*Evidence & Conclusion Ontology ID* [P3811]	CC0
*Relations Ontology* [Q28729320]	A collection of relations intended primarily for standardization across ontologies in the OBO Foundry and wider OBO library	http://purl.obolibrary.org/obo/ro.owl	*Relations Ontology ID* [P3590]	CC0
*Xenopus Anatomical Ontology* [Q42400040]	An ontology of the anatomy of the African clawed frog (Xenopus laevis).	http://purl.obolibrary.org/obo/xao.owl	*Xenopus Anatomical Ontology ID* [P4495]	CC-BY 3.0
*Cell Ontology* [Q55118285]	An ontology for cell types	http://purl.obolibrary.org/obo/cl.owl	*Cell Ontology ID* [P7963]	CC-BY 4.0
*Vaccine Ontology* [Q55118646]	An ontology for concepts related to vaccines and vaccination	http://purl.obolibrary.org/obo/vo.owl	*Vaccine Ontology ID* [P1928]	CC-BY 4.0
*BRENDA tissue/enzyme source* [Q81661549]	An ontology for the source of enzymes	http://purl.obolibrary.org/obo/bto.owl	*Brenda Tissue Ontology ID* [P5501]	CC-BY 4.0
*Hymenoptera Anatomy Ontology* [Q81661648]	An ontology of the anatomy of the Hymenoptera (bees, wasps, and ants)	http://purl.obolibrary.org/obo/hao.owl	*Hymenoptera Anatomy Ontology ID* [P9356]	CC0

In our exploration of license compatibility across the fifteen analyzed ontologies (Table 1), we uncovered a surprising trend: nine of them are governed by the Creative Commons Attribution (CC-BY) License, while only five share the same license as Wikidata (CC0). This discrepancy may be attributed to the fact that CC-BY licenses often grant exemptions for concept IDs, unlike statements and labels in OBO ontologies, which face limitations when directly imported into Wikidata [68]. This underscores the legal feasibility of aligning OBO ontologies with Wikidata, irrespective of their licenses, and leveraging Wikidata's wealth of information to enhance and sustain the analyzed ontologies. This aligns closely with the objectives of our current approach.

The parsing errors led to the discarding of five invalid ontologies, including Gender, Sex, and Sexual Orientation Ontology [Q97063846], Environment Ontology [Q31110555], Spider Ontology [Q42404539], Food Ontology [Q55118395], and Protein Ontology [Q55118584]. The details are available in Table 2. Two large-scale ontologies, namely the *Food Ontology* [69] and the *Protein Ontology* [70], have thousands of items and are crowdsourced and regularly synchronized based on various semantic resources. While OBO ontologies play a crucial role in organizing and standardizing biomedical semantic knowledge, they encounter various technical challenges that impact their effective utilization. For instance, the Food Ontology erroneously defines rdf:resource as http://www.w3.org/1999/02/22-rdf-syntax-ns#}resource instead of the correct http://www.w3.org/1999/02/22-rdf-syntax-ns#resource, which can hinder seamless parsing by computer programs. Similarly, the *Protein Ontology* faced an issue with its Secure Socket Layer (SSL) Certificate not being verifiable as of August 8th, 2023. However, it is important to note that these issues are not insurmountable. Addressing the SSL Certificate concern for the Protein Ontology simply requires renewing the certificate, a standard practice in maintaining web-based resources. Similarly, for other smaller ontologies within the OBO framework, such as the *Environment Ontology* and *Gender, Sex, and Sexual Orientation Ontology*, challenges like processing Unicode characters (e.g., ö in Jökulhlaup) and using deprecated IDs can be rectified through diligent maintenance efforts. Additionally, resolving web hosting errors, like the Forbidden HTTP Error 403 encountered in the Spider Ontology, contributes to enhancing accessibility and usability. It is worth acknowledging the substantial efforts of the OBO ontology curators and contributors in maintaining these resources. With a considerable number of active contributors dedicated to the upkeep of OBO ontologies [69,70], addressing technical issues and ensuring data integrity is not only feasible but also essential for enriching biomedical semantic knowledge. Moreover, recognizing that some of these issues may arise due to technical intricacies, such as unaligned caches, underscores the ongoing efforts required to optimize these resources effectively.

**Table 2:** Invalid OBO Ontologies linked to Wikidata (as of August 8th, 2023).

Ontology	Description	URI	Wikidata Property	Reasons for invalidity
*Gender, Sex, and Sexual Orientation Ontology* [Q97063846]	An ontology about gender identity and expression, sexual and romantic identity and orientation, and sexual and reproductive behavior	http://purl.obolibrary.org/obo/gsso.owl	*GSSO ID* [P9827]	**KeyError:** 'GSSO:008740′
*Environment Ontology* [Q31110555]	An ontology for environmental systems, components, and processes	http://purl.obolibrary.org/obo/envo.owl	*Environment Ontology ID* [P3859]	**ValueError:** invalid identifier: https://en.wikipedia.org/wiki/J÷kulhlaup
*Spider Ontology* [Q42404539]	Ontology for spider comparative biology including anatomical parts (e.g. leg, claw), behavior (e.g. courtship, combing) and products (i.g. silk, web, borrow) of The Tree of Life: Phylogeny of Spiders project	http://purl.obolibrary.org/obo/spd.owl	*Spider Ontology ID* [P4537]	**urllib.error.HTTPError:** HTTP Error 403: Forbidden
*Food Ontology* [Q55118395]	A simple lightweight ontology for publishing data about recipes	http://purl.obolibrary.org/obo/foodon.owl	*FoodOn ID* [P6767]	**KeyError:** '{http://www.w3.org/1999/02/22-rdf-syntax-ns#}resource'
*Protein Ontology* [Q55118584]	An ontology that provides an ontological representation of protein-related entities by explicitly defining them and showing the relationships between them	http://purl.obolibrary.org/obo/pr.owl	*PRotein Ontology ID* [P4926]	**urllib.error.URLError:** <urlopen error [SSL: CERTIFICATE_VERIFY_FAILED] certificate verify failed: self signed certificate in certificate chain (_ssl.c:997)>

When assessing the volume of the considered fifteen OBO ontologies, we found that all of them except the *Relations Ontology* have more than 1000 items as shown in Table 3. Eight ontologies have more than 5000 items. This is mainly due to crowdsourcing efforts and the long-term maintenance of the OBO ontologies by an active community [28]. Crowdsourcing allows for the collective input and collaboration of numerous individuals, often from diverse backgrounds and expertise, to contribute to the development and refinement of these ontologies [28]. By harnessing the collective intelligence of this community, the OBO ontologies can evolve and adapt over time to incorporate new knowledge, address emerging research areas, and improve their overall accuracy and completeness [28]. The active maintenance of these ontologies by the community ensures that they remain relevant and up-to-date in the rapidly advancing fields of biology and biomedicine. This ongoing effort involves tasks such as adding new terms, revising existing definitions, resolving inconsistencies, and incorporating feedback from users and domain experts. Through this collaborative process, the OBO ontologies can continue to serve as valuable resources for researchers, clinicians, and other stakeholders in the life sciences community [28].

**Table 3:** Coverage of OBO Ontology items in Wikidata (as of August 8th, 2023). Ontologies in Bold correspond to those qualifying for Wikidata-based semantic enrichment (Having 100 valid IDs or more in Wikidata).

Ontology	Ontology Items	Wikidata items	Missing IDs in Wikidata	Wrong IDs in Wikidata
***Sequence types and features ontology* [Q81661803]**	2632	178	2424	0
*Symptom Ontology* [Q81661810]	1013	849	1013	849^a^
***Gene Ontology* [Q135085]**	51241	43560	4092	0
***Disease Ontology* [Q5282129]**	13859	10618	3239	2
*Uberon* [Q7876491]	15823	6355	15823	6355^b^
***Human Phenotype Ontology* [Q17027854]**	17875	1854	16018	3
*Cell line Ontology* [Q21039006]	39080	33760	39080	33760^c^
***Mondo Disease Ontology* [Q27468140**]	26583	17736	8850	3
*Evidence and Conclusion Ontology* [Q28445410]	2056	12	2044	0
*Relations Ontology* [Q28729320]	28	28	28	28^d^
*Xenopus Anatomical Ontology* [Q42400040]	1775	273	1771	269^e^
*Cell Ontology* [Q55118285]	2961	2658	2955	2652^f^
*Vaccine Ontology* [Q55118646]	6510	65	6510	65^g^
***BRENDA tissue/enzyme source* [Q81661549]**	6569	228	6342	1
*Hymenoptera Anatomy Ontology* [Q81661648]	2596	5	2596	5^h^

a “SO:” Prefix missing.

b “UBERON:” Prefix missing.

c “CLO:” Prefix substituted by “CLO_”.

d “RO:” Prefix substituted by “RO_”.

e “XAO:” Prefix substituted by “XAO_”.

f “CL:” Prefix substituted by “CL_”.

g “VO:” Prefix substituted by “VO_”.

h “HAO:” Prefix missing.

However, only six ontologies have 100 valid item identifiers or more inside Wikidata (Bold in Table 3). Some of the ontologies have good coverage in Wikidata. Nevertheless, the IDs of their concepts in Wikidata do not match the ones in OBO ontologies. This is primarily attributed to the inadequate formatting of external identifiers in Wikidata, wherein PREFIX: is replaced by PREFIX_ (for example, in *Cell Line Ontology*) to conform to the URI formatting of the entities, or even PREFIX: is entirely omitted from the identifiers (as seen in *Symptom Ontology*). This can be due to some problems with the definition of Wikidata properties of the identifiers for these OBO ontologies [9] or to a deficiency in the settings of Mix'n’match [30]. Concerning the ontologies not significantly represented in Wikidata such as *Evidence and Conclusion Ontology*, this can be explained by the lack of a Wikidata bot that mass imports the data of these ontologies into Wikidata. Most of the significantly represented OBO ontologies have been imported into Wikidata thanks to Mix'n’match [1] and to Python bots [9].

The lack of integration of several OBO ontologies can be also explained by license incompatibility between several OBO Foundry ontologies and Wikidata. In fact, Integrating OBO ontologies with Wikidata indeed raises significant challenges due to differences in licensing. OBO ontologies typically use licenses like Creative Commons Attribution (CC-BY) [10], while Wikidata operates under the CC0 (Creative Commons Zero) license, which allows for maximal reuse and compatibility with other licenses [71]. The variance in licensing terms can pose obstacles for seamless integration and alignment between these resources. One of the primary concerns is the potential clash between the more permissive CC0 license of Wikidata and the restrictive terms of certain OBO ontologies [72]. For instance, if an OBO ontology employs a Share-Alike (SA) provision, requiring derivative works to be licensed under the same terms, integrating such data with Wikidata might be problematic, as Wikidata's CC0 license does not impose such requirements. This discrepancy may impede the transfer of data between these resources or lead to legal uncertainties regarding the licensing status of integrated datasets [72]. Furthermore, the integration process may also be hindered by differing standards for attribution and citation. OBO ontologies typically mandate specific attribution requirements, whereas Wikidata's CC0 license allows for attribution waivers, potentially complicating the establishment of proper attribution practices when combining data from these sources [72]. Addressing these licensing disparities is crucial for ensuring the legality, ethicality, and sustainability of data integration efforts between OBO ontologies and Wikidata. One possible approach is to negotiate agreements or waivers that reconcile conflicting licensing terms, allowing for harmonious integration while respecting the original authors' rights and licenses. Alternatively, developers may need to implement technical solutions, such as data transformation tools or middleware, to facilitate interoperability while ensuring compliance with relevant licensing obligations [73].

When assessing the six considerably represented ontologies in Wikidata, we find an efficient language representation of the labels of their elements covering more than ten languages as shown in Table 4. This language support for the names of the OBO ontology entities in Wikidata is mainly linked to the creation of articles about these biomedical items across language editions of Wikipedia [1,8]. This can also be due to importing information from multilingual resources like authority control databases [7] such as the *National Diet Library of Japan*. An interesting fact is the less efficient representation of languages in the glosses of the supported ontology entities in Wikidata, commonly known as Descriptions. This occurs despite the existence of web tools that allow to automatically add descriptions to Wikidata entities fulfilling a set of conditions. For example, *Wikidata Terminator*,^12^ developed by Magnus Manske, allows users to find the list of Wikidata items that have specific characteristics and that do not have descriptions using SPARQL. Also, Wwwyzzerdd,^13^ developed by BrokenSegue, allows the direct import of descriptions for a Wikidata item from the leads of corresponding Wikipedia articles. This is mainly related to the lack of awareness about these tools and the limited representation of specific language speakers inside the Wikidata community [1]. The need for language speakers to manually edit Wikidata is confirmed when seeing the number of significantly represented languages when covering the alternative names of Wikidata items, better known as aliases.

**Table 4:** Language representation of OBO Ontology items in Wikidata (as of August 8th, 2023).

Ontology	Labels (Languages >50 Labels)	Descriptions (Languages >50 Descriptions)	Aliases (Languages >100 aliases)	Represented Items
*Sequence types and features ontology* [Q81661803]	2521 (16)	890 (1)	1700 (4)	178
*Gene Ontology* [Q135085]	70769 (86)	89697 (27)	128336 (31)	43560
*Disease Ontology* [Q5282129]	88113 (132)	39941 (47)	94129 (51)	10618
*Human Phenotype Ontology* [Q17027854]	42185 (106)	11860 (37)	30486 (42)	1854
*Mondo Disease Ontology* [Q27468140]	99944 (125)	45886 (45)	115385 (50)	17736
*BRENDA tissue/enzyme source* [Q81661549]	5096 (31)	1578 (9)	2620 (6)	228

When seeing the representation of the items of the six considered OBO ontologies in ten selected languages, we find that all these languages cover a significant number of the covered entities as shown in Table 5. However, there is a bias of representation of entities per language where quite all the items support English, and less important coverage of items is provided in Cyrillic or non-European languages like *Russian*, *Chinese,* and *Arabic*. The better coverage of English is mainly due to the representation of the items in English inside the OBO Ontologies [28]. The bias towards European languages is mainly related to the better existence of the native speakers of these languages inside the Wikidata community [1]. That being said, the coverage of these languages in Wikidata can be very efficient to turn OBO ontologies into multilingual ones, even partially. The language data provided by Wikidata can be a good starting point for such a task when combined with other resources, particularly the ones developed by the World Health Organization and scientific societies like ICD-11, an international classification of diseases fully covering Arabic, English, French, Chinese, Russian, and Spanish [74].

**Table 5:** Language representation of the main labels of OBO Ontology items in Wikidata (as of August 8th, 2023): *English* (en), *French* (fr), *Spanish* (es), *German* (de), *Portuguese* (pt), *Dutch* (nl), *Chinese* (zh), *Arabic* (ar), and *Russian* (ru).

Ontology	en	fr	es	de	pt	nl	zh	ja	ar	ru	Items
*Sequence types and features ontology* [Q81661803]	178	85	70	79	57	63	86	89	72	76	178
*Gene Ontology* [Q135085]	43559	960	783	664	418	527	642	1600	574	724	43560
*Disease Ontology* [Q5282129]	10618	6375	3108	2632	1783	1607	2043	2151	2850	1777	10618
*Human Phenotype Ontology* [Q17027854]	1854	1660	1215	1130	966	837	1038	1034	1285	893	1854
*Mondo Disease Ontology* [Q27468140]	17736	9750	3533	3092	1862	1655	2177	2331	3252	1822	17736
*BRENDA tissue/enzyme source* [Q81661549]	228	162	156	148	145	137	123	108	103	102	228

In the analysis of the number of relations linking the Wikidata items aligned to the six considered OBO ontologies, we identify that most of these Wikidata relations are mainly ones linking entities from the same ontology as shown in Table 6. This is particularly seen in *Gene Ontology*, *Disease Ontology*, and *Mondo Disease Ontology*. Moreover, we find that a significant number of relations between Wikidata items representing OBO ontologies having a similar topic of interest like (*Disease Ontology*, *Human Phenotype Ontology*) and (*Disease Ontology*, *Mondo Disease Ontology*). This can partly be related to the better emphasis on taxonomic relations like *instance of* and *subclass of* in OBO ontologies [69,70]. We also find that the representation of the Wikidata relations that Wikidata can support between entities from different ontologies is distorted. For instance, Wikidata covers 12,593 genetic association [P2293] relations as of August 8th, 2023. 10550 of these associations involve items from *Disease Ontology*. However, none of them are linked to items from *Gene Ontology*. This can be explained by the lack of alignment between the Wikidata items about genes, particularly retrieved from *RefSeq* and other resources [9], and their corresponding IDs in *Gene Ontology*).

**Table 6:** Number of semantic relations between OBO ontology items as available in Wikidata (as of August 8th, 2023).

		Object item Ontology					
		*Sequence types and features ontology* [Q81661803]	*Gene Ontology* [Q135085]	*Disease Ontology* [Q5282129]	*Human Phenotype Ontology* [Q17027854]	*Mondo Disease Ontology* [Q27468140]	*BRENDA tissue/enzyme source* [Q81661549]
***Subject item Ontology***	*Sequence types and features ontology* [Q81661803]	82	43	1	0	2	0
	*Gene Ontology* [Q135085]	36	132555	20	4	15	12
	*Disease Ontology* [Q5282129]	2	59	22332	6014	26775	69
	*Human Phenotype Ontology* [Q17027854]	0	34	2973	1198	3777	13
	*Mondo Disease Ontology* [Q27468140]	6	73	24888	7022	40408	76
	*BRENDA tissue/enzyme source* [Q81661549]	0	14	19	6	18	55

When seeing the top Wikidata relation types between OBO ontology items, we confirm the domination of taxonomic relation types like *subclass of* [P279], *part of* [P361], *has part(s)* [P527], and *instance of* [P31] over the associations between the considered entities, particularly when the subjects and objects of the relations are from the same ontology, as shown in Table 7. These semantic relations are probably derived from the OBO ontologies themselves [69,70]. But, due to the crowdsourcing-based editing of Wikidata [1,8], some of them can be extracted from other ontological databases. It will be interesting to compare OBO ontology taxonomy with the Wikidata ontology for cross-enrichment and cross-validation purposes [31]. Further than taxonomic relations, Wikidata represents several non-taxonomic relations between OBO ontology items. When the relations link between entities from the same ontology, they range from generic relations trying to discriminate and disambiguate between close biomedical entities like *opposite of* [P461] and *different from* [P1889] to causality relations trying to reveal the mechanisms behind and manifestations of human body functioning, human diseases, and drug therapies like *regulates* [P128], *has cause* [P848], *symptoms and signs* [P780], and *connects with* [P2789]. When the Wikidata relations link between items from different OBO ontologies, the represented relation types are mainly ones related to the main topic of the subject ontology like *molecular function* [P680], *anatomical location* [P927], and *phenotype* [P6532]. In such a situation, the mass import of OBO relations can help diversify the relation types in Wikidata by covering different facets of the topics of the OBO ontologies [69,70]. Human editing and crowdsourcing in Wikidata can help enrich OBO relations by providing new statements derived from multiple resources [1,8].

**Table 7:** Top four semantic relation types between considered OBO ontology items as available in Wikidata (as of August 8th, 2023).

		Object item source	
		Same OBO ontology	Other OBO ontologies
***Subject item Ontology***	*Sequence types and features ontology* [Q81661803]	*subclass of* [P279]	*part of* [P361]
		50 relations	24 relations
		*part of* [P361]	*instance of* [P31]
		10 relations	6 relations
		*instance of* [P31]	*subclass of* [P279]
		10 relations	6 relations
		*opposite of* [P461]	*molecular function* [P680]
		4 relations	2 relations
	*Gene Ontology* [Q135085]	*subclass of* [P279]	*has part(s)* [P527]
		70064 relations	23 relations
		*instance of* [P31]	*subclass of* [P279]
		43545 relations	21 relations
		*regulates* [P128]	*regulates* [P128]
		9634 relations	16 relations
		*part of* [P361]	*anatomical location* [P927]
		8018 relations	12 relations
	*Disease Ontology* [Q5282129]	*subclass of* [P279]	*subclass of* [P279]
		19018 relations	24193 relations
		*instance of* [P31]	*instance of* [P31]
		2314 relations	5954 relations
		*symptoms and signs* [P780]	*symptoms and signs* [P780]
		620 relations	2739 relations
		*different from* [P1889]	*has phenotype* [P6532]
		136 relations	234 relations
	*Human Phenotype Ontology* [Q17027854]	*subclass of* [P279]	*subclass of* [P279]
		842 relations	5314 relations
		*symptoms and signs* [P780]	*instance of* [P31]
		234 relations	1137 relations
		*has cause* [P828]	*has cause* [P828]
		75 relations	206 relations
		*different from* [P1889]	*symptoms and signs* [P780]
		47 relations	139 relations
	*Mondo Disease Ontology* [Q27468140]	*subclass of* [P279]	*subclass of* [P279]
		29330 relations	25185 relations
		*instance of* [P31]	*instance of* [P31]
		11078 relations	4063 relations
		*symptoms and signs* [P780]	*symptoms and signs* [P780]
		760 relations	2727 relations
		*different from* [P1889]	*has phenotype* [P6532]
		123 relations	201 relations
	*BRENDA tissue/enzyme source* [Q81661549]	*part of* [P361]	*established from medical condition* [P5166]
		14 relations	40 relations
		*connects with* [P2789]	*development of anatomical structure* [P4843]
		10 relations	8 relations
		*has part(s)* [P527]	*medical condition* [P1050]
		9 relations	3 relations
		*subclass of* [P279]	*has part(s)* [P527]
		7 relations	2 relations

The integration of OBO and Wikidata represents a pivotal step towards fostering greater interconnectedness and accessibility within the realms of biological and biomedical knowledge, aligning closely with the core principles of linked data. By merging these two expansive repositories, the potential for cross-domain collaboration and data integration is substantially heightened, promising to enrich not only the understanding of biological systems but also their interplay with various other fields encapsulated within the vast expanse of Wikidata. However, the convergence of OBO with Wikidata is not without its challenges, chief among them being the meticulous replication of data and the implementation of robust version control mechanisms [75]. Ensuring the fidelity of replicated data across disparate platforms necessitates careful attention to detail, as discrepancies between OBO versions and corresponding revisions in Wikidata could undermine the reliability and utility of integrated information [75]. To navigate these complexities, intricate data transformation processes must be devised to harmonize the differing schemas and structures inherent to OBO and Wikidata. Moreover, stringent version control protocols must be established to track changes, resolve conflicts, and uphold the integrity of linked data representations amidst evolving ontologies and datasets [75]. Additionally, comprehensive metadata management strategies, encompassing thorough annotation and rigorous quality assurance measures, are imperative to provide crucial context, traceability, and reliability to integrated datasets [75].

### 4.2 Interaction between wikidata and MeSH keywords

**Fig. 3:**
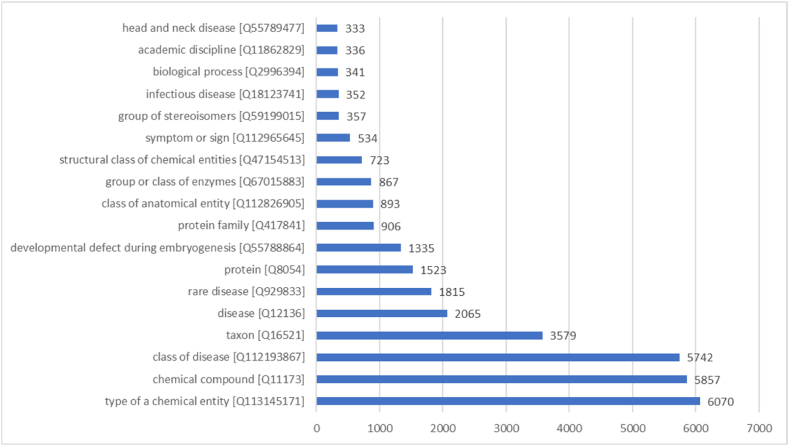
Top classes of the Wikidata items aligned to Medical Subject Headings as of August 8th, 2023 [SPARQL query: https://w.wiki/7EiQ].

As of August 8th, 2023, there are 38,703 Wikidata items aligned to Medical Subject Headings (MeSH) entities. These items represent different facets of the medical practice ranging from taxons and proteins to diseases and drugs as shown in Fig. 3. This number is comparable to one of the main headings in PubMed (30,454 concepts as in2023^14^). This can be explained by the existence of various projects to enrich Wikidata with multiple types of medical knowledge such as clinical trials [76], molecular biology data [9], and disease information [1]. This aligns with the overall distribution of biomedical classes in Wikidata [71]. As well, this is in part related to the representation of biomedical concepts across the language editions of Wikipedia [77]. Despite the interesting number of MeSH IDs in Wikidata, 3243 of them (8.3 %) are inaccurate, and 138 of them (0.3 %) are duplicates only corresponding to 65 MeSH IDs. As well, 5654 of the items (14.6 %) are not linked to other MeSH terms in Wikidata. This is mainly linked to the manual editing of Wikidata, which can cause minor inconsistencies [71]. The Wikidata community is invited to work on solving the inaccuracies in the semantic alignment between the open knowledge graph and MeSH.

**Fig. 4:**
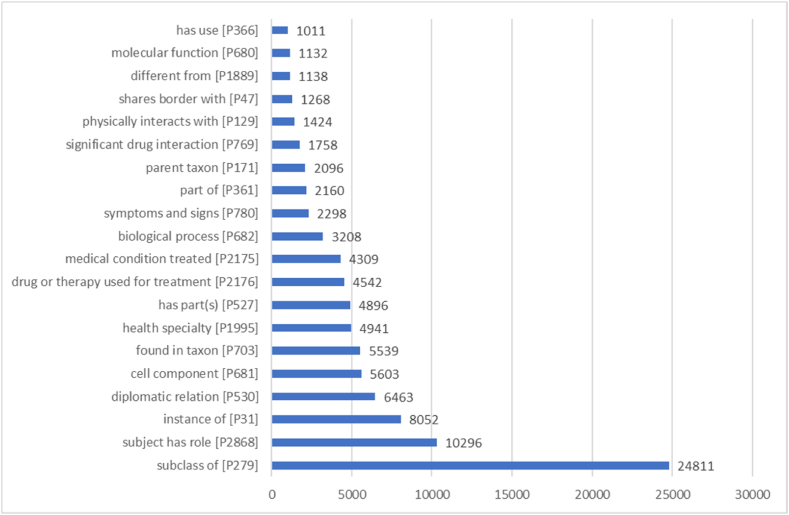
Top relation types between the Wikidata items aligned to Medical Subject Headings as of August 8th, 2023 [SPARQL query: https://w.wiki/7Eit].

Wikidata maintains 109,302 relations about entities linked to the MeSH database. 12,961 of them (11.8 %) cannot be verified as they involve Wikidata items with wrong MeSH Descriptor IDs. When seeing the types of the relations between MeSH terms in Wikidata, we find they are mostly dominated by taxonomic relation types such as *subclass of* [P279], *instance of* [P31], and *part of* [P361] as shown in Fig. 4. These types of relations are the main ones available in external resources like Medical Subject Headings (MeSH) [78] and Open Biological and Biomedical Ontologies [9,11]. They can also be easily derived from other Wikimedia projects, mainly Wikipedia through the analysis of its category graph and infoboxes [78]. Further than taxonomic relations, Wikidata also covers generic non-symmetric relations that can be used for a variety of purposes such as *subject has role* [P2868], *physically interacts with* [P129], *different from* [P1889], and *has use* [P366]. These relations can serve to disambiguate the entities and define their mechanisms of work [33]. As well, several non-biomedical non-symmetric relation types like *diplomatic relation* [P530] and *shares border with* [P47] are represented in Wikidata. These relations are not well discussed in PubMed publications as they have little to do with the medical practice [14]. 10.13039/100014337Furthermore, multiple biomedical relation types are supported by Wikidata thanks to the efforts of a wide community of computational biology [33] and medicine [71] editors in the knowledge graph. These relation types are mostly non-symmetric, like *cell component* [P681], *found in taxon* [P703], *health specialty* [P1995], *drug or therapy used for treatment* [P2176], *medical condition treated* [P2175] and *biological process* [P682]. The most important symmetric relation type in Wikidata is *significant drug interaction* [P769].

**Fig. 5:**
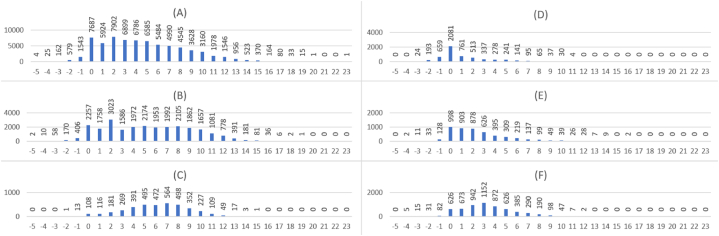
Wikidata relations by the down rounds of their pointwise mutual information values. (−10) values are not included in the bar plot: (A) *All relation types*, (B) *instance of* [P31], *subclass of* [P279] and *has part(s)* [P527], (C) *subject has role* [P2868], D) *diplomatic relation* [P530], E) *cell component* [P681] and *found in taxon* [P703], F) *health specialty* [P1995] and *drug or therapy used for treatment* [P2176].

When computing the pointwise mutual information of these relations, we find that their values range between −5.73 and 23.10 except for the relations having no MeSH associations in PubMed (24,800 relations - 22.7 %), receiving (−10) as a default PMI value. The distribution of the PMI values follows the Gauss law as shown in Fig. 5A [p-value for Kolmogorov-Smirov test <0.001], where most relations have PMI values from 0 to 6 with a peak at 2.0. As a result, we consider 2.0 as the threshold for PMI under which relations are not considered valid. The decision to use a threshold of 2.0 is based on prior research that employed different thresholds for semantic similarity and semantic relatedness. For semantic similarity, a threshold of 7.0 was utilized, while for semantic relatedness, a threshold of less than 3.0 was employed. These values were determined through the computation of Pointwise Mutual Information (PMI) using a previously processed large-scale corpus [79]. Notably, a previous study aimed to establish an effective PMI threshold by benchmarking against Rubenstein and Goodenough's dataset for semantic relatedness [80]. This study identified a threshold value of 2.0 as the most suitable for their purposes [81]. Breaking down the distribution of PMI values per relation type, we find quite a similar distribution for taxonomic (Fig. 5B), generic (Fig. 5C), and biomedical relation types (Fig. 5F) where most of the relations have a PMI that exceeds 2.0, confirming the relevance of considering 2.0 as the PMI threshold. However, we identify that non-biomedical (Fig. 5D) and computational biology (Fig. 5E) relations are mainly having PMI values around 0.0. This is mainly due to the lack of representation of non-biomedical knowledge in PubMed, as it is mainly a bibliographic database for biomedical scholarly publications [14].

When seeing the Wikidata semantic relations below the PMI threshold, we identified 15,925 out of 96,341 valid relations between items having accurate MeSH Descriptor IDs (16.5 %) as having a PMI below the threshold of 2, further than the 24,800 relations not found in PubMed. The assessment of the relation types behind these 40,725 deficient relations, as indicated in Table 8, highlights again that non-biomedical (e.g., *diplomatic relation* [P530]) and computational biology (e.g., *found in taxon* [P703] and *cell component* [P681]) relation types are hard to identify using PubMed because this database mainly includes bibliographic metadata about biomedical scholarly publications [14]. This is confirmed by the better identification of taxonomic (e.g., *subclass of* [P279]) and biomedical (e.g., *drug or therapy used for treatment* [P2176]) relation types using PubMed. The taxonomic (e.g., *instance of* [P31] and *has part(s)* [P527]) and biomedical (e.g., *symptoms and signs* [P780]) having an important rate of deficient relations (>50 %) are mainly linked to limitations in the human editing of Wikidata, causing the emergence of Wikidata relations not supported by PubMed-indexed biomedical scholarly publications [14].

**Table 8:** Top relation types per number of deficient relations between Wikidata items having accurate MeSH IDs.

Wikidata Property	Relations below PMI Threshold	Relations unavailable in PubMed	Deficient valid relations	Percentage among valid relations
*subclass of* [P279]	1714	4687	6401	29 %
*subject has role* [P2868]	238	5034	5272	59 %
*diplomatic relation* [P530]	3718	934	4652	73 %
*instance of* [P31]	1456	2830	4286	58 %
*found in taxon* [P703]	657	2065	2722	72 %
*health specialty* [P1995]	1105	1608	2713	57 %
*cell component* [P681]	1418	1200	2618	59 %
*has part(s)* [P527]	1492	995	2487	57 %
*symptoms and signs* [P780]	643	510	1153	53 %
*drug or therapy used for treatment* [P2176]	327	740	1067	29 %
*medical condition treated* [P2175]	310	702	1012	29 %

**Fig. 6:**
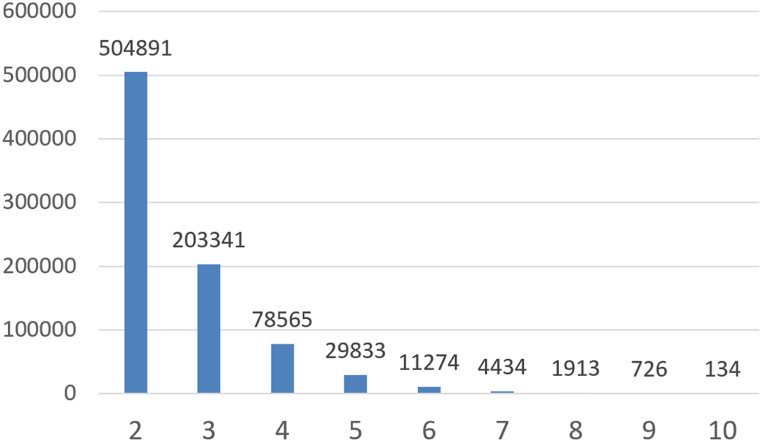
Distribution of the identified missing relations per the lower rounds of their PMI values.

After an extensive analysis of associations among the 5000 most common MeSH keywords in PubMed, our research uncovered 835,111 significant relationships that exhibited a Pointwise Mutual Information (PMI) score of 2 or higher. Notably, these relationships were conspicuously absent from Wikidata, underscoring a substantial body of biomedical knowledge that resides beyond the scope of the open knowledge graph [71]. Our findings demonstrated that the PMI values for these uncharted relationships consistently fell below 11, a trend we have depicted in Fig. 6. To delve into the specifics, we observed that 504,891 (approximately 60.5 %) of these relationships had PMI values less than 3, while 323,013 (around 38.7 %) possessed PMI values exceeding 3 but remaining strictly less than 7. A minor fraction of 7207 (about 0.8 %) achieved PMI values of 7 or higher. This distribution suggests that the majority of these associations are situated near the PMI threshold, highlighting the need for human verification given their relatively weak nature, as articulated [79]. In contrast, a minuscule fraction of non-existent relationships exhibited strong associations between the involved elements [79]. In such cases, these relationships may be deemed more substantial or significant, although they remain rare exceptions within the dataset. Such associations should also be subject to human verification. High PMI values do indicate that a relationship has been explored repeatedly in PubMed [82]. However, they do not guarantee the veracity of the relationship [83]. Multiple studies can produce negative results, challenging a given assumption [84].

After training the Dense Model to perform biomedical relation classification based on the *MiniMeSH2Matrix* dataset, we obtained an accuracy of 89.40 % and an F1-Score of 89.43 % for the superclass-based classification. As for the relation type-based classification, we got an accuracy of 75.32 % and an F1-Score of 73.51 %. The higher accuracy for the superclass-based classification is mainly due to its lower complexity, as opposed to relation type-based classification. In fact, the existence of multiple labels reduces the sample size per class, affecting how much a machine-learning algorithm is trained to recognize every label [85]. The accuracy rates for *MiniMeSH2Matrix* are higher than the ones for the original *MeSH2Matrix* dataset that achieved an accuracy of 83.09 % and an F1-Score of 82.94 % for the superclass-based classification and an accuracy of 70.78 % and an F1-Score of 66.90 % for the relation type-based classification [14]. This is in part due to the elimination of non-biomedical relations that cannot be discriminated against by the qualifiers of MeSH keywords [14]. As well, this can be due to the reduction of the number of classes to 56 relation types (195 for *MeSH2Matrix*) and 3 superclasses (5 for *MeSH2Matrix*) [14,85]. The better accuracy for the *MiniMeSH2Matrix*-based supervised classification proves the ability of Integrated Gradients (Sundarajan et al., 2017) to recognize the contributing factors to a classification while reducing the size of the classification dataset by 92.48 % from 2.74 GB (for *MeSH2Matrix*) to 210.8 MB (for *MiniMeSH2Matrix*).

**Fig. 7:**
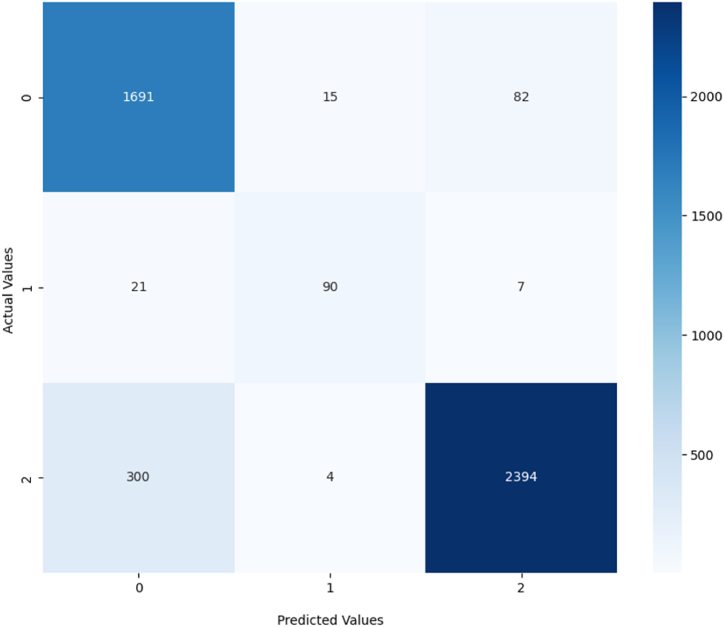
Confusion Matrix for the superclass-based classification: *Taxonomic* (0), *Biomedical Symmetric* (1), and *Biomedical Non-Symmetric* (2).

The study of the confusion matrix for the superclass-based supervised classification reveals a lack of differentiation between taxonomic and biomedical non-symmetric relations, contributing to 89.04 % (382 out of 429) of the mistakenly assigned superclasses, as shown in Fig. 7. This can be explained by the similarity of features between the matrices of taxonomic relations and the ones of biomedical non-symmetric relations [14]. Both relations are asymmetric and that is why contributing features to the classification of semantic relations as taxonomic and biomedical non-symmetric can overlap [14]. Probably, splitting the matrices into two sets where the features having a subject qualifier equal to the subject qualifier are put apart can solve the problem as taxonomic relations mainly link between entities from the same class while biomedical non-symmetric relations mainly relate between entities from different parent classes [14]. Another important finding is that the classification of biomedical symmetric relations is accurate at a precision rate of 76.2 % (90 out of 118) and a recall rate of 82.6 % (90 out of 109). This is a better efficiency for the classification of the biomedical symmetric relations than using the original *MeSH2Matrix* dataset [14]. This is mainly linked to the elimination of non-biomedical symmetric relations that share a lot of common features with biomedical symmetric relations [14]. This better accuracy occurs despite the class imbalance against biomedical symmetric relations as they only represent 2.6 % (801 out of 30,693) of the represented matrices in the *MiniMeSH2Matrix* dataset. Class imbalance negatively affects the accuracy of the classification of the marginalized classes [86]. Consequently, increasing the number of supported biomedical symmetric relations can be very useful to increase the accuracy of the classification of this type of semantic relations.

**Fig. 8:**
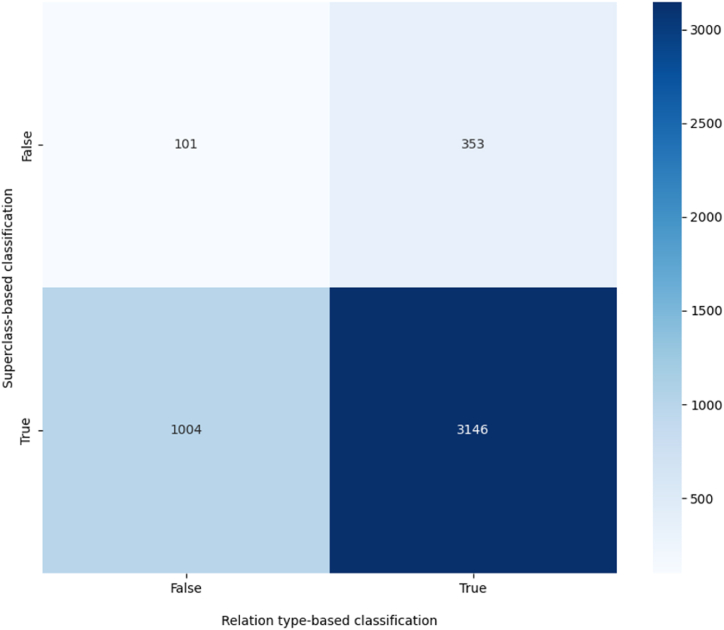
Matrix of agreement between the accurate predictions for superclass-based classification and the ones for relation type-based classification.

The analysis of the agreement between the accurate predictions for superclass-based classification and the ones for relation type-based classification identifies that 68.3 % of the matrices receive accurate predictions for both superclass-based classification and relation type-based classification (3146 out of 4604 test matrices) as shown in Fig. 8. Among the matrices that receive inaccurate predictions in at least one of the two classifications (31.7 % of all matrices), only 6.9 % of them (101 out of 1458 test matrices) receive inaccurate predictions in both classifications (2.2 % of all matrices). This proves the efficiency of the comparison of the parent class of the relation type assigned to matrices with the superclasses assigned to the same matrices to identify not accurately classified matrices without supervision. In fact, this technique will identify 90.85 % (1004 out of 1105 test matrices) of the mistakenly assigned relation types and 77.75 % (353 out of 454 test matrices) of the false superclasses. This goes in line with previous works on using taxonomic relations, such as *instance of*, *subclass of*, and *part of*, to identify the ability of supervised classification algorithms to assign the labels having a common parent class [87], to align predicted labels to corresponding expected labels in multi-label classification [88], to augment the output of the multi-label classification by adding the parts (e.g., *eye* and *nose*) of a recognized entity (e.g., *face*) when they are not identified by the machine-learning algorithm [89], and to split multi-label classification tasks into sets of independent mono-label classification tasks [90].

When utilizing the Dense Model to classify MeSH relations within a dataset encompassing 835,111 associations derived from Pointwise Mutual Information, an intriguing discovery emerged. Specifically, our analysis uncovered that a substantial portion of these connections, amounting to 579,412 relations (69.3 %), did not have designated relation types. Among the unclassified relations, a significant proportion, specifically 91.1 % (528,005 relations), remain unclassified because their subjects and objects cannot be associated with MeSH qualifiers necessary for creating classification matrices. Additionally, 51,407 relations (8.9 % of unclassified relations) do not have assigned relation types due to conflicting results from both the superclass-based and relation type-based classification methods. This observation aligns with the research findings of Turki et al. [14], who reported that 42.6 % of the semantic relations between MeSH Keywords in Wikidata cannot be represented by their own classification matrices. This phenomenon can be ascribed to potential inaccuracies in the assignment of relation types or the classification of certain relations within broader categories. It is crucial to underscore that this occurrence may also be attributed to the limited scope of biomedical information available in Wikidata. Addressing this issue may necessitate the introduction of new properties to enrich the biomedical data model of the knowledge graph, particularly using https://www.wikidata.org/wiki/Wikidata:Property_proposal [1]. To delve deeper into understanding the nature of these relations that do not have Wikidata-supported relation types, a specific method was employed. We extracted the initial letter of the MeSH Tree Code (P672) associated with both the subject and object of each unassigned association. This initial letter signifies the category of the respective concept it represents, as elaborated in Table 9. An analysis of the MeSH tree categories reveals that the primary unsupported relations establish connections between entities from the same categories, mainly *D – Chemical and Drugs* (56,846 relations), *C – Diseases* (18,167 relations), *N – Health Care* (13,860 relations), and *G – Biological Sciences* (11,996 relations). These relations are likely to be taxonomic ones due to the inclusion of their subjects and objects in the same class [14]. These relations are more likely to be taxonomic ones from a probabilistic point of view [14]. However, they can also be biomedical symmetric, meaning that the entities have a mutual or reciprocal relationship (e.g., comorbidity, where two or more diseases occur together in the same time) or asymmetric, meaning that the entities have a one-way relationship (e.g., causality, where one entity influences or affects another) [91] Furthermore, we have detected noteworthy associations between *D - Chemical and Drugs* and other classes, particularly *G - Biological Sciences*, *E - Analytical, Diagnostic and Therapeutic Techniques and Equipment*, *A - Anatomy*, and *C - Diseases*. This shows that drug classes are defined not only by their chemical structures or mechanisms of action but also by their therapeutic applications and impacts on various aspects of human health [92]. However, Wikidata seems to cover only a limited set of drug information, mainly used properties such as medical condition treated [P2175], drug or therapy used for treatment [P2176], or significant drug interactions [P769] [14].

**Table 9:** Unassigned associations according to the MeSH categories of their subjects and objects.

Subject MeSH Tree Category	Object MeSH Tree Category	Relations	Rate
D – Chemical and Drugs	D – Chemical and Drugs	56,846	9.8 %
G – Biological Sciences	D – Chemical and Drugs	19,919	3.4 %
C – Diseases	C – Diseases	18,167	3.1 %
D – Chemical and Drugs	G – Biological Sciences	13,863	2.4 %
N – Health Care	N – Health Care	13,860	2.4 %
E − Analytical, Diagnostic and Therapeutic Techniques and Equipment	D – Chemical and Drugs	13,857	2.4 %
A – Anatomy	D – Chemical and Drugs	12,155	2.1 %
G – Biological Sciences	G – Biological Sciences	11,996	2.1 %
C – Diseases	D – Chemical and Drugs	10,752	1.8 %
D – Chemical and Drugs	C – Diseases	9702	1.7 %

Upon examining the primary Wikidata relation types assigned to discovered associations, we observed that the *subclass of* relation [P279] is the most prevalent, accounting for 93,197 relations (36.4 % of the total). This is followed by the *instance of* relation [P31], with 26,820 relations, and the *cell component* relation [P681], with 16,655 relations. Additionally, we identified various other relation types, encompassing drug-related and cellular biology relations, among others as shown in Table 10. The lack of *instance of* [P31] and *subclass of* [P279] relations serves as a clear indicator of the incomplete nature of the Wikidata taxonomy. This observation underscores the notion that the Wikidata knowledge graph is not as densely populated as one might expect [93]. The underrepresentation of these key relationships reflects a deficiency in the comprehensive organization of data within the Wikidata framework, highlighting the need for ongoing efforts to enrich and refine its taxonomy. The limited coverage of several types of biomedical relations highlights the need for substantial improvements in this domain. This shortfall not only blocks our ability to extract comprehensive knowledge from the Wikidata platform in the context of biomedical research [1] but also highlights the importance of ongoing collaborative efforts to enhance the representation of vital biomedical information [71].

**Table 10:** Top relation types per number of newly identified relations based on Pointwise Mutual Information.

Relation Type	Relations	Rate of classified relations
*subclass of* [P279]	93,197	36.4 %
*instance of* [P31]	26,820	10.5 %
*cell component* [P681]	24,212	9.5 %
*biological process* [P682]	16,655	6.5 %
*found in taxon* [P703]	16,624	6.5 %
*health specialty* [P1995]	10,378	4.1 %
*anatomical location* [P927]	9834	3.8 %
*medical condition treated* [P2175]	7729	3.0 %
*drug or therapy used for treatment* [P2176]	6953	2.7 %
*significant drug interaction* [P769]	6432	2.5 %
***Other***	36,865	14.4 %

When adding references from PubMed to 1000 random unsupported statements in Wikidata using the PubMed search engine and the Best Match sorting algorithm, we found that most of the unsupported relations in Wikidata are mainly taxonomic and non-biomedical ones as shown in Table 11. This is mainly explained by the mass import of biomedical relations from open biological and biomedical ontologies, attributing the added Wikidata statements to their source ontologies [9,11]. Non-biomedical Wikidata relations not having references are mainly the ones involving countries such as *diplomatic relation* [P530], *shares border with* [P47], and *country* [P17]. This is mostly due to the exclusive involvement of bibliographic metadata about biomedical scholarly publications in PubMed [94]. Taxonomic relations lacking references correspond to the most represented relation types such as *subclass of* [P279], *has part(s)* [P527], *instance of* [P31], and *part of* [P361]. These relation types are represented in PubMed and can be accurately identified through leveraging MeSH keywords [14]. They can also be easily extracted from open biological and biomedical ontologies [9,11]. The lack of reference support for taxonomic relations in Wikidata can be explained in part by the lack of the full mass import of open biological and biomedical ontologies into Wikidata despite the technical easiness of doing this, particularly thanks to the availability of a wide community of contributors of computational biology information to Wikidata that developed bots and tools for mass importing OBO ontologies into Wikidata [9,11]. This is also linked to the existence of inaccurate taxonomic relations in Wikidata that have been mistakenly added by the Wikidata Community and that should be removed [14].

**Table 11:** Top relation types by rate of unsupported relations.

Relation Type	Rate of unsupported relations
*subclass of* [P279]	29.4 %
*diplomatic relation* [P530]	9.4 %
*health specialty* [P1995]	9.3 %
*has part(s)* [P527]	8.8 %
*instance of* [P31]	7.5 %
*shares border with* [P47]	3.0 %
*part of* [P361]	2.9 %
*symptoms and signs* [P780]	2.8 %
*parent taxon* [P171]	2.1 %
*facet of* [P1269]	2.1 %
*country* [P17]	1.7 %
*has cause* [P828]	1.4 %
*has use* [P366]	1.4 %
*Gram staining* [P2597]	1.3 %
*subject has role* [P2868]	1.2 %
*drug or therapy used for treatment* [P2176]	1.0 %

When we undertook the task of assigning references to these unsupported relations using the PubMed search engine, a significant revelation emerged. It was discovered that a substantial 45.8 % of the references lacked corresponding evidence in PubMed, as can be seen in Table 12. The underlying reasons for this outcome are multi-faceted. One contributing factor is the inherent inaccuracy found in several Wikidata relations. This inaccuracy can be attributed, in part, to the collaborative nature of Wikidata as a knowledge graph that is subject to continual editing and refinement [14]. Consequently, the imperative for human validation of these relations becomes even more pronounced. Another factor influencing this discrepancy can be traced back to the relatively recent discovery of certain biomedical facts that find support within Wikidata. It is important to recognize that these facts often require time to transition from discovery to widespread acceptance, a process that involves evidence collection, synthesis, and the authoring of systematic reviews [95]. This latency period can delay the endorsement of these facts through review papers. The dynamic landscape of medical research continually ushers in new insights and revelations, but the comprehensive establishment of references with a high level of evidence demands time and rigorous analysis [95].

**Table 12:** Unsupported relations per number of assigned references from PubMed.

Number of assigned references	0	1	2	3+
Rate of unsupported statements	45.8 %	8.1 %	4.1 %	42.0 %

The revelation that 45.8 % of unsupported relations within Wikidata lack corroboration from PubMed underscores a significant disparity that warrants urgent attention. This discrepancy not only raises concerns regarding the reliability of Wikidata but also highlights potential implications for its users, spanning from researchers to developers and beyond. The necessity for a thorough investigation into the root causes of these inconsistencies cannot be overstated. Factors contributing to this gap may originate from various sources, including lapses in data quality control mechanisms, disparities in the reliability and credibility of sourced information, and inherent semantic ambiguities that may arise during data interpretation and integration [31]. To address these challenges and bolster the reliability of Wikidata, multifaceted strategies are imperative. Strengthening data validation processes through enhanced automation and integration with reputable sources like PubMed can help identify and rectify inaccuracies [96]. Additionally, fostering greater community engagement and establishing robust peer review mechanisms can leverage collective expertise to address inconsistencies [31]. Standardizing documentation and guidelines for data entry and interpretation can mitigate semantic ambiguities, promoting greater consistency across entries [97]. Continuous monitoring and updates to reflect the latest research findings and align with reputable sources are essential for ensuring data accuracy and relevance over time [97].

In contrast to this, an intriguing statistic emerged. Approximately 42.0 % of the unsupported relations demonstrated the potential for alignment with three or more references, drawing from the capabilities of the PubMed search engine and the Best Match algorithm. These references, rooted in the exploration of MeSH keywords [98], ostensibly bolster the associations in question. However, it is imperative to discern that these references, while indicative of the associations' existence in scientific literature, do not inherently validate the accuracy of the relations themselves. Instead, they affirm that these associations have been subjects of scrutiny among medical scientists [84]. A critical juncture for human validation arises, necessitating a meticulous examination to ascertain the alignment of each reference with the corresponding relation. Moreover, an intriguing subset of unsupported relations, comprising 12.2 %, occupies a distinctive position. These particular relations can only be substantiated by a limited range of one or two review papers. This observation introduces another layer of complexity to the task of assigning references to unsupported relations. This scenario can be intrinsically linked to the temporal aspect of biomedical discoveries. This paucity of references does not inherently denote inaccuracies within the relation itself; rather, it illuminates the nascent stage of scientific exploration and documentation [95]. As time unfolds and further research is conducted, the body of evidence substantiating these relations is anticipated to expand. While the quantity of references may be constrained in this category, their significance remains undiminished. These references constitute the initial endeavors of medical scientists to delve into and authenticate these connections. However, the relatively limited number of references buttresses the necessity for an enduring commitment to validation and verification, particularly within burgeoning fields where knowledge is still emerging.

The examination of the number of allocated references for each relation type highlights a notable bias in how pertinent resources are distributed among specific relation types, as depicted in Table 13. Specifically, the utilization of the PubMed search engine and the Best Match algorithm demonstrates a primary efficacy in associating pertinent references with taxonomic relation types (*subclass of* [P279] and *part of* [P361]), general relation types (*facet of* [P1269], *has cause* [P828], and *has use* [P366]), along with a restricted subset of other asymmetrical relation types (namely, *country* [P17] and *Gram staining* [P2597]). This information will likely be included in the definitions and explanations of biomedical concepts as involved in the introductory notes and main body of biomedical reviews [99]. Surprisingly, the attribution of references to biomedical relation types, like *health specialty* [P1995], *symptoms and signs* [P780], *parent taxon* [P171], and *drug or therapy used for treatment* [P2176], is limited (<50 %). This is mainly due to the fact that literature reviews are meant to provide a broad overview of its main topic [99]. Despite the recommendation for the creation of systematic reviews with a narrow scope for evidence-based clinical reasoning [100], it is not common that a review studies a single association between a disease and a specific drug or symptom. A review mostly compares the effect of a set of drugs in curing a disease or the significance of multiple symptoms to identify a medical condition to choose the right path for patient management [101]. Considering this, expanding our approach to cover biomedical articles in PubMed can be sufficient to solve the lack of assignment of reviews as references to unsupported biomedical relations in Wikidata.

**Table 13:** Unsupported relations per number of assigned references from PubMed for top relation types.

Number of assigned references	0	1	2	3+
*subclass of* [P279]	28.6 %	4.1 %	3.7 %	**63.6 %**
*diplomatic relation* [P530]	**57.4 %**	13.8 %	3.2 %	25.5 %
*health specialty* [P1995]	**67.7 %**	11.8 %	5.4 %	15.1 %
*has part(s)* [P527]	**65.9 %**	3.4 %	1.1 %	29.5 %
*instance of* [P31]	**64.0 %**	8.0 %	4.0 %	24.0 %
*shares border with* [P47]	**40.0 %**	20.0 %	10.0 %	30.0 %
*part of* [P361]	17.2 %	3.4 %	6.9 %	**72.4 %**
*symptoms and signs* [P780]	**67.9 %**	7.1 %	3.6 %	21.4 %
*parent taxon* [P171]	**52.4 %**	0.0 %	9.5 %	38.1 %
*facet of* [P1269]	19.0 %	9.5 %	0.0 %	**71.4 %**
*country* [P17]	29.4 %	17.6 %	11.8 %	**41.2 %**
*has cause* [P828]	14.3 %	14.3 %	0.0 %	**71.4 %**
*has use* [P366]	**71.4 %**	0.0 %	0.0 %	28.6 %
*Gram staining* [P2597]	15.4 %	15.4 %	0.0 %	**69.2 %**
*subject has role* [P2868]	**91.7 %**	0.0 %	0.0 %	8.3 %
*drug or therapy used for treatment* [P2176]	**60.0 %**	10.0 %	0.0 %	30.0 %
Others	**43.5 %**	11.6 %	5.4 %	39.5 %

## 5 Conclusion

This research presents a comprehensive framework aimed at enhancing Wikidata's role as an open and collaborative knowledge graph. It harnesses the rich information from Open Biological and Biomedical Ontologies (OBO) and MeSH keywords found in PubMed scholarly publications. Utilizing SPARQL, a potent query language for RDF knowledge graphs, semantic knowledge is extracted from Wikidata to enhance OBO ontologies. However, the study exposes gaps in the semantic alignment between OBO and Wikidata, underscoring the necessity for both automated methods and human intervention to rectify this disparity. Nevertheless, the research showcases Wikidata's potential for enriching and expanding OBO ontologies, particularly in multilingual contexts. The collaboration between the OBO Community and the Wikidata Community offers mutual benefits by leveraging crowdsourcing efforts and long-term maintenance. OBO's expertise enhances Wikidata with domain-specific ontology structures, while Wikidata's vast dataset enriches OBO ontologies. This collaboration fosters interoperability, data integration, and interdisciplinary research, advancing knowledge organization in biological and biomedical sciences. MeSH keywords prove invaluable for biomedical relation extraction and classification, bolstered by innovative employment of pointwise mutual information. Integrated Gradients further refine the classification process, enhancing accuracy levels for superclass-based and relation type-based classifications. Investigating the relationship between parent classes and assigned relation types provides a tool for identifying misclassified relations, indicating the practicality of this approach in refining classification outcomes. Moreover, exploration of unsupported relations within Wikidata using MeSH keywords highlights potential inconsistencies stemming from collaborative editing. MeSH keywords' fact-checking role, validated efficiently through PubMed references, reinforces their significance in ensuring accurate and reliable semantic relations.

Looking forward, the research sets the stage for several promising avenues of exploration. Addressing semantic alignment gaps between OBO and Wikidata may involve advanced automated techniques combining natural language processing, ontological mapping, and machine learning. Incorporating OBO ontologies covered by the CC0 license but not yet represented in Wikidata has the potential to enhance the breadth and depth of medical information available within the Wikidata repository. Collaborative workflows integrating machine algorithms with human expertise hold promise for enhancing the knowledge graph's accuracy and comprehensiveness. Further refinement of classification processes through additional context-based attributes could enhance accuracy. Expanding Wikidata's multilingual capabilities and implementing quality assurance mechanisms are crucial for enhancing global usability and data integrity. Investigating dynamic updating mechanisms and alternative validation strategies beyond MeSH keywords could ensure the knowledge graph's currency and robustness. Additionally, embedding privacy, bias mitigation, and accountability measures within the knowledge graph is essential for ethical data management.

## Ethical considerations

Our research paper adheres to stringent ethical considerations despite its focus on the analysis of publicly available resources utilizing open-source code. We confirm that no human subjects have been involved in this study, and all data utilized primarily consist of biomedical semantic data and metadata extracted from scholarly publications. While the absence of human subjects mitigates certain privacy concerns, we remain committed to upholding ethical principles such as data integrity, transparency, and algorithmic accountability. By emphasizing the ethical use of openly accessible data and open-source methodologies, we aim to ensure the credibility, reproducibility, and societal impact of our research outcomes.

## Data availability statement

The source data and code for this research work are released under the GNU General Public License at https://doi.org/10.6084/m9.figshare.24438184. A testbed playground is available at https://drive.google.com/file/d/1xZxw541mbbbB_byPD1bkbZbrcgyL2J6G/view?usp=sharing.

## CRediT authorship contribution statement

**Houcemeddine Turki:** Writing – review & editing, Writing – original draft, Visualization, Validation, Supervision, Software, Resources, Project administration, Methodology, Investigation, Funding acquisition, Formal analysis, Data curation, Conceptualization. **Khalil Chebil:** Software, Resources, Methodology, Investigation, Formal analysis, Data curation, Conceptualization. **Bonaventure F.P. Dossou:** Writing – review & editing, Writing – original draft, Supervision, Software, Resources, Methodology, Investigation, Formal analysis, Data curation, Conceptualization. **Chris Chinenye Emezue:** Writing – review & editing, Software, Methodology, Investigation, Formal analysis, Data curation. **Abraham Toluwase Owodunni:** Writing – review & editing, Software, Investigation, Formal analysis, Data curation. **Mohamed Ali Hadj Taieb:** Validation, Supervision, Project administration, Funding acquisition, Conceptualization. **Mohamed Ben Aouicha:** Validation, Supervision, Project administration, Funding acquisition, Conceptualization.

## Declaration of generative AI and AI-assisted technologies in the writing process

During the preparation of this work the authors used ChatGPT, a large language model-based chatbot maintained by OpenAI, for language proofreading. After using this tool/service, the authors reviewed and edited the content as needed and took full responsibility for the content of the publication.

## Declaration of competing interest

The authors declare the following financial interests/personal relationships which may be considered as potential competing interests:Houcemeddine Turki reports financial support was provided by 10.13039/100011049Wikimedia Foundation Inc. If there are other authors, they declare that they have no known competing financial interests or personal relationships that could have appeared to influence the work reported in this paper.

## Footnotes

2 https://wikidata.org/w/api.php.

3 https://github.com/LeMyst/WikibaseIntegrator.

4 https://www.wikidata.org/wiki/Wikidata:Database_download.

5 https://meta.wikimedia.org/wiki/Mix%27n%27match.

6 https://pronto.readthedocs.io/.

7 The dataset is available at https://github.com/SisonkeBiotik-Africa/MeSH2Matrix.

8 Every qualifier is attributed an identifier as they appear in this list where *analysis* receives 0 as an ID, *pathology* receives 1 as an ID, and so on.

9 Query: ("<Keyword_1>/∗"[Majr:NoExp]) AND ("<Keyword_2>/∗"[Majr:NoExp]).

10 The list is available at http://obofoundry.org/.

11 New Wikidata properties can be added using https://www.wikidata.org/wiki/Wikidata:Property_proposal.

12 https://wikidata-terminator.toolforge.org/#/.

13 https://www.wikidata.org/wiki/Wikidata:Wwwyzzerdd.

14 An Update about MeSH in 2023: https://www.nlm.nih.gov/oet/ed/mesh/2023/mesh_highlights.html.
