# Supplementary material for: A framework for integrating biomedical knowledge in Wikidata with open biological and biomedical ontologies and MeSH keywords
Source: Heliyon. 2024 Sep 27;10(19):e38448. doi: 10.1016/j.heliyon.2024.e38448 (PMC11471508; doi:10.1016/j.heliyon.2024.e38448)
Supplement: Multimedia component 1 [file mmc1.docx]

**Appendices**

**Table S1:** SPARQL queries for the interaction between Wikidata and Open Biological and Biomedical Ontologies. *Disease Ontology ID* [P699] and *Gene Ontology ID* [P686] are used as examples in T2-4.

| Task | Description | SPARQL query |
| --- | --- | --- |
| T1 | Extracting the list of OBO ontologies having a valid URI and a Wikidata property | SELECT * WHERE {  #Finding the URI for OBO Foundry Ontologies  ?ontologywdt:P31 wd:Q324254;  wdt:P361 wd:Q4117183;  wdt:P2888?uri.  #Finding the Wikidata Property for the Ontology IDs  ?ontologywdt:P1687?prop.  } |
| T2 | Retrieving the items having an ID in a given ontology from Wikidata | SELECT * WHERE {  ?subjectwdt:P699?subjectID.  } |
| T3 | Extracting the labels (*rdfs:label*), descriptions (*schema:description*), and aliases (*skos:altLabel*) of every Wikidata item corresponding to an ontology entity | SELECT?subjectID?prop ?object (LANG(?object) as ?lang) WHERE {  ?subjectwdt:P699?subjectID.  VALUES?prop {rdfs:label schema:description skos:altLabel}  ?subject ?prop ?object.  } |
| T4 | Extracting the semantic relations between the ontology entities and other entities from the same ontology or other OBO ontologies, as available in Wikidata | SELECT?subjectID ?prop ?objectIDWHERE {  ?subjectwdt:P699?subjectID.  ?objectwdt:P686?objectID.  ?subject ?prop ?object.  } |

**Table S2:** SPARQL queries for the interaction between Wikidata and MeSH Keywords.

| Task | Description | SPARQL query |
| --- | --- | --- |
| L1 | Extracting the list of all the Wikidata items corresponding to MeSH terms | SELECT?subject ?subjectIDWHERE {  ?subjectwdt:P486?subjectID.  } |
| L2 | Extracting the list of Wikidata relations between MeSH terms | SELECT?subject ?subjectID ?prop ?object ?objectIDWHERE {  ?subjectwdt:P486?subjectID.  ?objectwdt:P486?objectID.  ?subject ?prop ?object.  } |
| L3 | Identifying 10k Wikidata relations between MeSH items lacking references | SELECT?subject ?subjectID ?prop ?object ?objectIDWHERE {  ?subject wdt:P486?subjectID.  ?objectwdt:P486?objectID.  ?subject ?prop ?statement.  FILTER(!(regex(str(?prop), "http://www.wikidata.org/prop/direct/" ) ))  FILTER(regex(str(?prop), "http://www.wikidata.org/prop/" ) )  ?statement ?p ?object.  FILTER(regex(str(?p), "http://www.wikidata.org/prop/statement/" ) )  FILTER NOT EXISTS { ?statementprov:wasDerivedFrom?derivedFrom .}  }  LIMIT 1000 |
